# Antenatal dexamethasone for early preterm birth in low-resource countries

**DOI:** 10.1056/NEJMoa2022398

**Published:** 2020-10-23

**Authors:** WHO ACTION Trials Collaborators

## Abstract

**BACKGROUND:**

The safety and efficacy of antenatal glucocorticoids for women at risk of preterm birth in low-resource countries is unknown.

**METHODS:**

We conducted a multicountry, randomized trial involving pregnant women at risk of preterm birth between 26 weeks 0 days and 33 weeks 6 days of gestation. Participants were assigned to intramuscular dexamethasone or identical placebo. Primary outcomes were neonatal death, any baby death (stillbirth or neonatal death), and composite possible maternal bacterial infection outcome. We applied superiority hypothesis for the infant primary outcomes and non-inferiority hypothesis for the maternal primary outcome.

**RESULTS:**

The trial was stopped at the second interim analysis for benefits. We randomized 2852 women (and their 3070 babies) from 29 secondary and tertiary level hospitals across Bangladesh, India, Kenya, Nigeria, and Pakistan. Neonatal death occurred in 278 of 1417 infants (19.6%) in the dexamethasone group and 331 of 1406 infants (23.5%) in the placebo group (relative risk, 0.84; 95% confidence interval [CI] 0.72 to 0.97; P=0.03). Any baby death occurred in 393 of 1532 infants (25.7%) in the dexamethasone group and 444 of 1519 infants (29.2%) in the placebo group (relative risk, 0.88; 95% CI 0.78 to 0.99; P=0.04). Possible maternal bacterial infection did not differ between dexamethasone and placebo groups (4.8% vs. 6.3%, relative risk, 0.76; 95% CI 0.56 to 1.03), a finding consistent with noninferiority (P=0.002). Early neonatal death, severe respiratory distress at 24 hours, neonatal hypoglycemia at 6 hours, resuscitation at birth, and use of continuous positive airway pressure were lower in the dexamethasone group. Adverse events did not differ significantly between the groups.

**CONCLUSIONS:**

Antenatal dexamethasone treatment of women at risk of early preterm birth in low-resource countries resulted in a significantly lower risk of neonatal death and any baby death, and no increase in possible maternal bacterial infection. (Funded by Bill and Melinda Gates Foundation; Australia and New Zealand Clinical Trials Registry number ACTRN12617000476336; Clinical Trials Registry-India number, CTRI/2017/04/008326)

## BACKGROUND

Preterm birth is a leading cause of newborn and under-five mortality globally.^1^ Infants born preterm are also at increased risk of a wide range of short- and long-term respiratory, infectious, metabolic and neurological morbidities, with higher risks for those born during the early preterm period.^2,3^

Antenatal glucocorticoids have long been promoted as the key intervention for reducing preterm infant mortality and morbidity based on trials largely conducted in high-resource countries.^4,5^ The generalizability of this evidence to low-resource settings was, however, called into question when in 2015 a large population-based trial in six low-resource countries showed that efforts to scale up antenatal glucocorticoids can lead to harm.^6,7^ Scaling up of glucocorticoids in the study did not confer mortality benefit for preterm infants, and unexpectedly led to increase in stillbirth, neonatal death, and maternal infection at the population level. These safety concerns reopened the debate about the lack of rigorous evidence on safety and efficacy of antenatal glucocorticoids in low-resource countries.^8,9^

Based on these considerations, the World Health Organization (WHO) recommended in 2015 that antenatal glucocorticoids should only be used in settings where certain conditions – accurate gestational age (GA) assessment, imminent preterm birth, absence of maternal infection, and adequate childbirth and preterm newborn care – can be met.^10,11^ The guideline panel and an expert panel subsequently convened by WHO identified efficacy trials in hospitals in low-resource countries as a research priority, in order to resolve this controversy and guide clinicians and policymakers on how best to use antenatal glucocorticoids.^8,9^ We conducted a randomized trial to assess the safety and efficacy of dexamethasone when given to women at risk of early preterm birth, in hospitals in low-resource countries.

## METHODS

### Trial design and oversight

We designed a multicountry, multicenter, individually-randomized, parallel-group, double-blind, placebo-controlled trial – the WHO ACTION-I (**A**ntenatal **C**orticos**T**eroids for **I**mproving **O**utcomes in preterm **N**ewborns) trial – to compare intramuscular (IM) dexamethasone with identical placebo for women at risk of imminent preterm birth. We conducted the trial at 29 secondary and tertiary level hospitals across six study sites in Bangladesh, India, Kenya, Nigeria and Pakistan. The trial protocol has been published previously.^12^ It was approved by the relevant ethics committees and regulatory agencies in each country, and WHO Ethics Review Committee. WHO was the trial sponsor. A steering group comprising a trial co-ordinating unit, principal investigators, and technical advisors, provided oversight for the trial. Dexamethasone sodium phosphate and matching placebos were procured from Fresenius Kabi/Labesfal, Portugal and packaged and shipped to study sites by Ivers-Lee CSM, Switzerland. Fresenius Kabi/Labesfal had no role in study design, data collection, analysis, interpretation, writing of the manuscript, or the decision to publish. OT Oladapo, JP Vogel, R Bahl and G Piaggio (of the trial co-ordinating unit) are responsible for the accuracy and completeness of the data and analyses and the fidelity of this report to the protocol.

### Study setting

Study hospitals were selected through a standardized assessment of maternal and newborn healthcare services (Table S1), to ensure that the WHO antenatal glucocorticoid treatment criteria could be reasonably met.^9^ To optimize trial procedures, ultrasound systems (Philips HD5, Netherlands), continuous positive airway pressure (CPAP) machines (Diamedica, Sweden), pulse oximeters (Masimo, Switzerland) and glucometers were procured for all hospitals. Standardized trainings were provided to all research and clinical staff.

### Screening and recruitment

Pregnant women (with confirmed live fetuses) who were at risk of preterm birth between 26 weeks 0 days and 33 weeks 6 days were eligible for inclusion. Inclusion criteria were: birth planned or expected in the next 48 hours (following preterm prelabour rupture of membranes, spontaneous labour, or provider-initiated preterm birth). GA was determined by earliest ultrasound or one performed at admission. Women were excluded if they had: clinical signs of severe infection; major congenital fetal anomalies; concurrent or recent (within the past two weeks) use of systemic glucocorticoids; participation in another trial; or contraindication to steroids. Written informed consent was obtained before randomization.

### Randomization and treatment

Participants were randomly assigned (1:1 ratio) to a course of IM injections of either 6 mg dexamethasone or placebo administered every 12 hours, to a maximum of four doses, or until hospital discharge or birth. Women were eligible for a repeat course if they had not given birth after seven completed days but still met inclusion criteria. The repeat course was identical to the first course, and the same as the initial allocation.

Site-stratified individual randomization with balanced permuted blocks of size 10 were used. The computer-generated randomization sequence was prepared centrally at WHO. All sites received serially numbered identical treatment packs containing 4mg/mL ampoules of dexamethasone or placebo for two full courses. Trial participants, care providers, and investigators were not aware of group assignments.

Women received allocated study treatment immediately after randomization. Clinical care was according to local guidelines. Follow up was conducted until 28 days after birth or death (whichever came first). Trained research staff collected data during hospital admission(s) and through community visits.

### Study outcomes

There were three primary outcomes: neonatal death (death of a liveborn within 28 completed days of life); any baby death (stillbirth or neonatal death); and a composite outcome for possible maternal bacterial infection, defined as maternal fever (≥ 38 °C) or clinically suspected or confirmed infection, for which therapeutic antibiotics were used. We hypothesised that the use of dexamethasone would result in a reduction in neonatal death and any baby death without increasing the risk of maternal infection. Therefore, we applied a superiority hypothesis to neonatal death and any baby death outcomes, and a non-inferiority hypothesis to maternal infection outcome. Secondary outcomes include maternal and newborn mortality and morbidity, and process of care outcomes. Definitions of all outcomes are provided in the Supplementary Appendix.

All study-related information was stored securely at study sites. Data were double-entered into a web-based, data management platform, and centrally managed by Centro Rosarino Estudios Perinatales, Argentina. Independent monitors performed source data verification according to a protocol.

### Statistical analysis

We estimated that 6018 women needed to be recruited to detect a reduction of 15% or more in neonatal death, from 25% to 21.3%, in a two-sided 5% significance test with 90% power and 10% loss to follow up. The estimated sample size would provide over 80% power at the 2.5% significance level to detect if dexamethasone is non-inferior to placebo for the maternal infection outcome, within a non-inferiority margin of 1.25 on the relative scale, and assuming a 10% baseline rate of maternal infection.

Primary analyses were based on intention-to-treat (ITT), analyzing all participants with outcome data available, and corrected for multiplicity of primary outcomes. The dexamethasone arm was compared against the placebo arm for the primary outcomes using relative risk with 95% confidence intervals, based on a logistic model with a binomial distribution and the log link to obtain relative risks. The stratifying variable, study hospital, was included in the model, as well as a clustering feature for multiple births for neonatal outcomes. For continuous variables, means and standard deviations or medians, quartiles and interquartile range by group were reported. Treatment groups were compared using mean or median differences and 95% confidence intervals based on a general linear model that included study site as stratifying variable. Separate models were fitted for each of the primary and secondary outcomes.

Prespecified subgroup analyses of the primary outcomes were performed based on whether preterm birth was planned, GA at first dose, number of fetuses, study site, time from first dose to birth, mode of birth, and use of tocolytics before birth. We further analysed the effect of time of first dose to birth on treatment effect using a logistic model, including GA at first dose and number of doses in the model.

Results for all secondary outcomes and subgroup analyses are presented as point estimates and 95% confidence interval without correction for multiple comparisons. All models were fitted using SAS Software version 9.4 (SAS Institute Inc., Cary, NC, USA).

Accruing data were monitored, in confidence, by the Data Safety Monitoring Board (DSMB) and three interim analyses were planned. The DSMB terms of reference were that they should inform the steering group chair if, in their view, there was proof beyond doubt that treatment with dexamethasone is indicated or contraindicated based on statistical or clinical considerations, practical issues, or external new information. The DSMB considered the Haybittle-Peto stopping rule^13^ for the primary infant mortality outcomes, as statistical guidance for their recommendation. After the second interim analysis of 2304 women and 2536 infants with complete follow-up of primary outcomes, the DSMB decided to unblind the trial and recommended the trial be stopped for infant mortality benefits, and strong evidence of a graded dose-response effect. Recruitment was stopped across all sites on 21 November 2019 and all ethics committees and regulatory authorities were informed. The funder had no role in the decision to stop the trial.

## RESULTS

### Characteristics of participants

**Figure 1:**
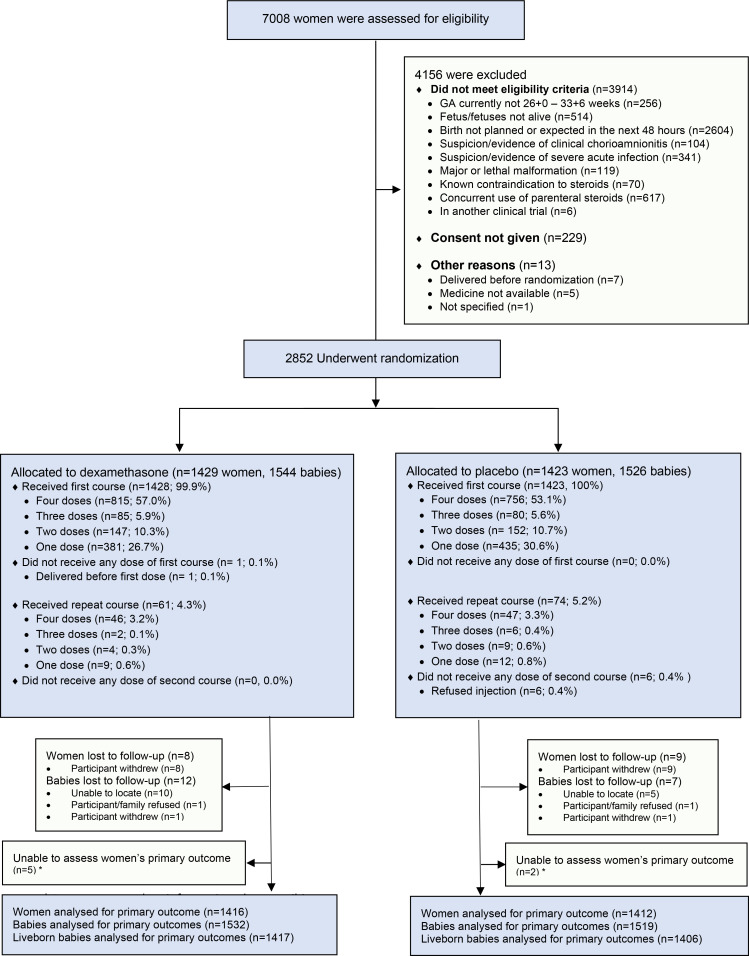
Screening, enrolment, randomization, and follow-up

Of the 7008 women who were screened for eligibility, 2852 women were randomized (1429 women to the dexamethasone group and 1423 women to the placebo group) from December 2017 through November 2019 (Figure 1). The most common reason for non-eligibility was that birth was not planned or expected in the next 48 hours. Birth occurred before 37 weeks for 90.0% of infants in the dexamethasone group and 90.8% of infants in the placebo group. Over 99% of randomized women and infants completed follow-up. The dexamethasone and placebo groups were similar at baseline (Table 1, Table S2).

**Table 1:** Characteristics of women at trial entry

Characteristic	Dexamethasone (N=1429)	Placebo (N=1423)
**Clinical assessment of imminent preterm birth at trial entry – no. (%)**		
**Spontaneously-initiated preterm birth**	874 (61.2)	858 (60.3)
Preterm prelabour rupture of membranes	455 (31.8)	388 (27.3)
Spontaneous preterm labour	419 (29.3)	470 (33.0)
**Provider-initiated preterm birth**	555 (38.8)	565 (39.7)
**Mean (± SD) gestational age at trial entry**	30.8 (2.0)	30.7 (2.0)
**Maternal age (yr) – mean (SD)**	27.5 (5.8)	27.5 (5.9)
**No. of fetuses in the current pregnancy – no. (%)**		
Single	1295 (90.6)	1290 (90.7)
Twin	125 (8.7)	129 (9.1)
Higher order multiples	9 (0.6)	4 (0.3)
**Nulliparity**	529 (37.0)	549 (38.6)
History of preterm birth – no. (%) *	177 (12.4)	188 (13.2)
Obstetric conditions currently present – no. (%) **		
Gestational diabetes	22 (1.5)	15 (1.1)
Pre-eclampsia or eclampsia	275 (19.2)	326 (22.9)
Gestational hypertension (excl. preeclampsia or eclampsia)	75 (5.2)	68 (4.8)
Oligohydramnios (known or suspected)	336 (23.5)	310 (21.8)
Polyhydramnios (known or suspected)	19 (1.3)	30 (2.1)
Intrauterine growth restriction (known or suspected)	94 (6.6)	95 (6.7)
Abruptio placentae	49 (3.4)	40 (2.8)
Placenta praevia	115 (8.0)	110 (7.7)
Other obstetric hemorrhage	66 (4.6)	42 (3.0)
No obstetric condition	616 (43.1)	592 (41.6)
**Medication administered prior to randomization – no. (%)**		
Tocolytic	251 (17.6)	267 (18.8)
Magnesium sulfate for neuroprotection	141 (9.9)	179 (12.6)

* Only among women with a previous pregnancy;

** Women may have had more than one condition; There was no significant difference between treatment groups at an experimentwise error rate of 5%; All characteristics of women at trial entry are available in Table S2.

### Compliance with allocated treatment

All women except one received at least one dose of their allocated treatment (Figure 1). A total of 815 of 1429 women (57.0%) in the dexamethasone group and 756 of 1423 women (53.1%) in the placebo group received all four doses of study medication in the first course. The repeat course was used in 61 women in the dexamethasone group and 74 women in the placebo group, of whom 46 and 47 women received four doses, respectively. The most common reason for non-administration of a scheduled dose was that birth had occurred between doses.

### Primary outcomes

There were 278 (19.6%) neonatal deaths among 1417 liveborn infants in the dexamethasone group and 331 (23.5%) neonatal deaths among 1406 liveborn infants in the placebo group (relative risk 0.84; 95% confidence interval [CI], 0.72 to 0.97; P=0.03) (Table 2). We determined that 25 women would need to be treated with dexamethasone to prevent one neonatal death (95% CI, 14 to 110). Any baby death was also significantly lower in the dexamethasone group than in the placebo group (25.7% vs 29.2%, relative risk 0.88; 95% CI 0.78 to 0.99; P=0.04).

**Table 2:** Primary outcomes

Primary outcome	Dexamethasone n/N (%)	Placebo n/N (%)	Relative risk (95% CI) *	P-value^§^
Neonatal death	278/1417 (19.6)	331/1406 (23.5)	0.84 (0.72-0.97)	0.03
Any baby death	393/1532 (25.7)	444/1519 (29.2)	0.88 (0.78-0.99)	0.04
Possible maternal bacterial infection ^ǂ^	68/1416 (4.8)	89/1412 (6.3)	0.76 (0.56-1.03)	0.002^¶^

* Relative risk and 95% CI, calculated from modelling, adjusting for study sites and taking into account the clustering due to multiple births; CIs are also adjusted for multiplicity;

§ P-value adjusted for multiplicity for the three primary outcomes using the False Discovery Rate approach;

¶ P-value for non-inferiority for possible maternal bacterial infection;

ǂ Defined as occurrence of maternal fever of ≥ 38 ^O^C or clinically suspected or confirmed infection, for which therapeutic antibiotics were used. Suspected or confirmed infection included obstetric infection (chorioamnionitis, postpartum endometritis, or wound infection) or non-obstetric infection (respiratory tract infection [pneumonia, pharyngitis, sinusitis or similar], urinary tract infection (excluding pyelonephritis), pyelonephritis, acute cholecystitis or other system infection), captured during hospital admission/s only.

Possible maternal bacterial infection occurred in 68 (4.8%) of 1416 women in the dexamethasone group and in 89 (6.3%) of 1412 women in the placebo group (relative risk, 0.76; 95% CI 0.56 to 1.03; P=0.002 for non-inferiority), a result consistent with noninferiority at the prespecified margin of 1.25 (Table 2). Based on per-protocol population, possible maternal infection occurred in 63 (4.5%) of 1393 women in the dexamethasone group and in 89 (6.4%) of 1385 women in the placebo group (relative risk, 0.70; 95% CI 0.51 to 0.96, with the same conclusion on non-inferiority as ITT analysis. Multiple imputations for missing values^14^ yielded identical results for all primary outcomes (Table S3).

**Figure 2:**
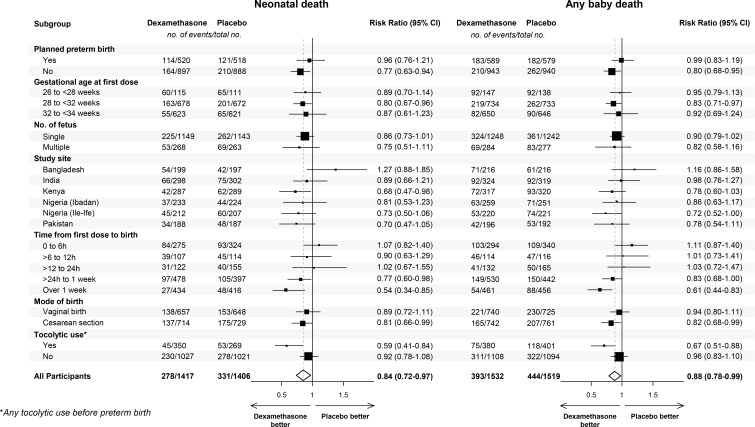
Neonatal primary outcomes by subgroups

The treatment effects on the primary outcomes based on subgroup analyses are shown in Figure 2, and Figure S1. Figures S2 shows the relative risks for dexamethasone versus placebo as a function of time from first dose to birth, for different GAs at first dose. The trend for the effect size to increase with time from first dose to birth, and GA at first dose from 26 to 32 weeks, is apparent. A post hoc analysis of the causes of neonatal death showed that neonatal death caused by respiratory distress syndrome was lower in the dexamethasone group (Table S4).

### Neonatal secondary outcomes

As shown in Table 3, early neonatal death was lower in the dexamethasone group, but there was no difference between groups for stillbirth. Severe respiratory distress at 24 hours and hypoglycemia at 6 hours were lower in the dexamethasone group but no differences were observed in the overall rates of severe respiratory distress and hypoglycemia measured within the first week of life. There was no difference in neonatal sepsis or other morbidities between groups. Major resuscitation at birth and use of CPAP were lower in the dexamethasone group. Table S5 shows that median duration of oxygen therapy was shorter and parenteral antibiotic use was longer in the dexamethasone group. Other secondary and process of care outcomes were similar between the groups (Table S5, Table S6).

**Table 3:** Secondary maternal and neonatal outcomes

Outcome	Dexamethasone n/N (%)	Placebo n/N (%)	Relative risk (95% CI)
**Neonatal outcome**			
Stillbirth	115/1544 (7.5)	113/1526 (7.4)	1.00 (0.78-1.30)
Early neonatal death (≤7 days)	218/1417 (15.4)	268/1406 (19.1)	0.81 (0.68-0.96)
Severe respiratory distress* • At 24 h	116/1245 (9.3)	141/1223 (11.5)	0.81 (0.64-1.03)
	34/1122 (3.0)	58/1065 (5.5)	0.56 (0.37-0.85)
Neonatal sepsis	183/1284 (14.3)	197/1264 (15.6)	0.92 (0.76-1.11)
Hypoglycemia * • At 6 h • At 36 h	301/1242 (24.2)	328/1217 (27.0)	0.91 (0.80-1.04)
	92/1224 (7.5)	123/1194 (10.3)	0.73 (0.56-0.95)
	54/1035 (5.2)	62/999 (6.2)	0.85 (0.60-1.21)
Major resuscitation at birth	101/1382 (7.3)	144/1383 (10.4)	0.70 (0.55-0.88)
Use of oxygen therapy*	726/1429 (50.8)	756/1413 (53.5)	0.95 (0.88-1.02)
Use of CPAP*	265/1429 (18.5)	337/1413 (23.9)	0.78 (0.67-0.90)
Use of mechanical ventilation*	83/1284 (6.5)	103/1264 (8.2)	0.79 (0.59-1.05)
Use of parenteral therapeutic antibiotics for 5 days or more ^ǂ^	527/1245 (42.3)	494/1175 (42.0)	1.00 (0.91-1.10)
Admission to a special care unit	905/1287 (70.3)	897/1268 (70.7)	0.99 (0.94-1.04)
**Maternal outcomes**			
Maternal death	5/1429 (0.4)	4/1423 (0.3)	1.23 (0.33-4.57)
Maternal fever	78/1417 (5.5)	70/1406 (5.0)	1.10 (0.80-1.50)
Chorioamnionitis	17/1429 (1.2)	18/1423 (1.3	0.93 (0.48-1.80)
Endometritis	5/1429 (0.4)	3/1423 (0.2)	1.65 (0.39-6.92)
Wound infection	8/1429 (0.6)	15/1423 (1.1)	0.53 (0.22-1.25)
Non-obstetric infection	34/1429 (2.4)	42/1423 (3.0)	0.81 (0.52- 1.26)
Therapeutic antibiotics	68/1427 (4.8)	89/1422 (6.3)	0.76 (0.56-1.03)
Any antibiotic use	1205/1353 (89.1)	1216/1355 (89.7)	1.00 (0.97-1.02)

* Measured during initial postnatal hospitalization only, until death, discharge or completed day 7 (whichever came first); h, hours; CPAP, continuous positive airway pressure;

ǂ parenteral therapeutic antibiotics for 5 days or more, even if interrupted, excluding neonates who died before 5 completed days; referral for treatment not presented because of very few events.

### Maternal secondary outcomes

There were no between-group differences in the rates of the maternal secondary outcomes (Table 3). Five maternal deaths occurred in the dexamethasone group and four in the placebo group. Overall antibiotic use was high, but therapeutic use was less frequent in both groups. Maternal readmission was rare in both groups. The median duration of hospital stay was 8 days in both groups (Table S5).

### Adverse events

Serious adverse events (SAE) among women did not differ significantly between the groups (1.1% vs. 1.1%, P=0.99) (Table S7). Besides neonatal deaths and morbidities reported as secondary outcomes, no other neonatal SAE were reported.

## DISCUSSION

In this hospital-based randomized trial in low-resource countries, we found that dexamethasone administration to women at risk of early preterm birth reduced neonatal deaths without increasing maternal infection. Dexamethasone had no impact on stillbirth but the findings for several secondary outcomes including early neonatal death, severe respiratory distress, use of major neonatal resuscitation and CPAP, were consistent with overall results for neonatal deaths by 28 days. The reduction in neonatal deaths was possibly mediated through a decrease in respiratory distress syndrome. These clinical benefits were observed despite 45% of women receiving less than four doses of their allocated medication. Mortality reduction appeared to increase with tocolysis and duration of fetal exposure to dexamethasone.

Our findings are generally consistent with the results of meta-analyses of 22 existing trials mostly conducted in high-resource settings, which found substantial reductions in neonatal death among infants of women treated with glucocorticoids.^4^ Our results provide the much needed evidence on the beneficial effects of glucocorticoids on reducing neonatal mortality in low-resource settings, and further strengthen the scarce body of evidence from low- and middle-income countries (LMICs).^15-19^

Consistent with previous trials in LMICs,^15-19^ dexamethasone did not increase the risk of maternal and neonatal infection, an important concern in our trial setting where the baseline risks of peripartum and neonatal infections are high.^20-22^ The lack of differential effect on overall rates of neonatal hypoglycemia, and the finding of reduced risk of early hypoglycemia with dexamethasone, were however unexpected, given that animal and pharmacokinetic studies suggest that neonatal hypoglycemia is a potential complication of standard doses of dexamethasone.^23^ Our observation is contrary to the results of Antenatal Late Preterm Steroids trial, where betamethasone administration to women at risk of preterm birth between 34 weeks 0 days and 36 weeks 5 days gestation increased the incidence of neonatal hypoglycemia by 60%.^24^ Whether this finding reflects differential effects of glucocorticoids on early versus late preterm infants is unclear. The literature on the direction of neonatal glycemic status following glucocorticoid administration in early preterm birth has not been consistent.^25,26^

This is the largest placebo-controlled trial on the efficacy and safety of antenatal glucocorticoids in low-resource countries to date. To increase generalizability of the findings, we applied eligibility criteria that were more inclusive than previous trials, assessed neonatal death according to the standard definition, and carefully selected hospitals that could reasonably meet minimum preconditions for glucocorticoid use in low-resource countries. The loss to follow up of trial participants and attrition of primary outcome data were very low despite community follow-up. The trial was limited by the challenges in standardizing maternal and neonatal care across study sites and the need for third trimester ultrasound GA confirmation for a substantial proportion of the participants.

The promotion of antenatal glucocorticoids in clinical practice, which was hitherto suboptimal in low-resource countries,^27^ was challenged by the serious concerns raised by Antenatal Corticosteroid Trial (ACT) publication in 2015.^6,28^ Those concerns have now been allayed by ACTION-I, a hospital based placebo-controlled trial, fundamentally different from ACT, which was a cluster trial of an implementation strategy. Key differences between ACT and ACTION-I trials relate to identification of women eligible for dexamethasone administration, and the standards of available maternal and newborn care. ACTION-I hospitals selected appropriate patient population for treatment (through assessment by obstetric physicians and GA verification by ultrasound) and provided minimum standards of care, measures that were largely not implemented in ACT. The measures in ACTION trial accounted for 90% of infants exposed to dexamethasone being born within the preterm period, minimizing over-treatment, and may explain the overall reduction in neonatal death. In comparison, only 16% of infants exposed to dexamethasone in ACT intervention clusters had birthweight less-than-5th-percentile (a proxy for preterm birth), highlighting substantial over-treatment, and consequent lack of mortality benefit in small babies, and overall harm. Appropriate patient selection and provision of minimum standard of care are thus critical to achieving benefits and preventing potential harms from glucocorticoids and should be incorporated into future implementation strategies.

Although we see no need for further placebo-controlled trials of antenatal glucocorticoids before 34 weeks, research gaps remain. There is no certainty about the optimal dosing regimen^29,30^ or the safety and efficacy of late preterm glucocorticoids,^31^ particularly in low-resource countries. The role of tocolytics in safely delaying early preterm birth for maximal benefits of ACS administration also merits further investigation.

In conclusion, antenatal dexamethasone treatment resulted in a significantly lower risk of neonatal death and any baby death than did placebo, without any evidence of harm to women or newborns. The policy challenges are to ensure much wider use of glucocorticoids in similar low-resource settings that have, where necessary, adapted to the standards of care used in ACTION: ultrasound assessment of GA, identification of imminent preterm birth (expected within 24-48 hours), appropriate management of birth, and a minimum package of neonatal care that includes access to oxygen and CPAP.

## DECLARATIONS

### Ethics approval and consent to participate

The study has been reviewed and approved by the WHO Ethics Review Committee, and all relevant institutional review boards.

### Competing interests

The authors declare that they have no competing interests.

### Funding

This trial was primarily funded by the Bill and Melinda Gates Foundation (Grant OPP1136821). Additional support was provided by UNDP/UNFPA/UNICEF/WHO/World Bank Special Programme of Research, Development and Research Training in Human Reproduction (HRP), Department of Sexual and Reproductive Health and Research; and Department of Maternal, Newborn, Child, Adolescent Health, and Ageing, of the World Health Organization, Geneva, Switzerland.

### Authors' contributions

This trial was initially conceived during a meeting convened by WHO, held in Geneva on 12-13 November 2015. JPV, OTO, AMG and RB co-ordinated the writing of the study protocol, with input from the country principal investigators and the technical advisory group. GP prepared the statistical analysis plan and led all statistical analysis with support from JC and statistical programming team. The trial steering group reviewed and interpreted the final data at a workshop convened by WHO. The first drafts for various sections of the manuscript were prepared by five writing subgroups drawn from the trial steering group. OTO consolidated the first draft, which was then reviewed and revised critically for intellectual content by all authors. All authors approved the final version and approved the manuscript for publication. The manuscript represents the views of the named authors only.
