# Supplementary material for: Antenatal dexamethasone for early preterm birth in low-resource countries
Source: N Engl J Med. Author manuscript; Available in PMC 2020 Dec 24. (PMC7660991; doi:10.1056/NEJMoa2022398)
Supplement: Supplementary file 1 [file NEJM-2020-2022398-s1.pdf]

Supplementary Appendix

This appendix has been provided by the authors to give readers additional information about their work.

Supplement to: WHO ACTION Trial Collaborators. Antenatal dexamethasone for early preterm birth in low-resource countries.

Table of Contents

WHO ACTION Trial Collaborators....2
Statistical methods....6
Sample size....6
Statistical analysis....6
Data safety monitoring....7
DSMB rationale for stopping the trial....8
Assessing confounders and effect modifiers of neonatal primary outcomes....10
Results....11
Neonatal death....11
Any baby death....12
Primary and secondary outcome definitions....13
Supplementary figures and tables....18
Figure S1: Possible maternal bacterial infection by subgroups....18
Figure S2. Relative risks of dexamethasone vs. placebo according to time from first dose to birth....19
Table S1. Characteristics of ACTION-I trial hospitals....21
Table S2. Characteristics of women at trial entry....36
Table S3. Primary outcomes with multiple imputation of missing values*....38
Table S4. Cause-specific neonatal mortality....38
Table S5. Other secondary maternal and neonatal outcomes....39
Table S6. Other secondary maternal and neonatal outcomes (contd.)....41
Table S7. Adverse events....42
Summary of the procedures to determine the final cause of neonatal death....43
Procedures relating to ultrasound assessments....43
Obstetric ultrasound for gestational age assessment....44
Neonatal transcranial ultrasound intraventricular haemorrhage assessment....45
References....47

WHO ACTION Trial Collaborators

- **Trial Co-ordinating Unit**
  - **UNDP/UNFPA/UNICEF/WHO/World Bank Special Programme of Research, Development and Research Training in Human Reproduction (HRP), Department of Sexual and Reproductive Health and Research, World Health Organization, Geneva, Switzerland:** Olufemi T. Oladapo MD, MPH, FWACS, Joshua P. Vogel BMedSci, MBBS, PhD, Gilda Piaggio MSc, PhD (Statistician), My Huong Nguyen MD, PhD (Data Manager), Fernando Althabe MD, MSc. (from 2019), A. Metin Gülmezoglu MD, PhD (2015-2019)
  - **Department of Maternal, Newborn, Child, Adolescent Health, and Ageing, World Health Organization, Geneva, Switzerland:** Rajiv Bahl MD, PhD, Suman PN Rao MBBS, MD, DM (from 2019), Ayesha De Costa MD, PhD (from 2019), Shuchita Gupta MBBS, MD, MPH, PhD (from 2019)
- **Bangladesh site:**
  - **Johns Hopkins Bloomberg School of Public Health, Baltimore, USA:** Abdullah H. Baqui MBBS, MPH, DrPH, (Principal Investigator), Rasheda Khanam MBBS, MPH, PhD
  - **Bangabandhu Sheikh Mujib Medical University, Shahbag, Dhaka, Bangladesh:** Mohammod Shahidullah MBBS, FCPS (Principal Investigator), Saleha Begum Chowdhury MBBS, FCPS (Principal Investigator)
  - **Projahnmo Research foundation, Banani, Dhaka, Bangladesh:** Salahuddin Ahmed MBBS, Nazma Begum MA, Dip. Comp. Sc, Arunangshu Dutta Roy MBBS (from 2018), M. A. Shahed MBBS (from 2018), Iffat Ara Jaben MBBS, MPH (from 2018)
  - **Centre for Woman and Child Health, Ashulia, Savar, Dhaka, Bangladesh:** Soofia Khatoon MBBS, FCPS, MHPEd, Anjuman Ara MBBS, FCPS (from 2018)
  - **Sylhet M. A. G. Osmani Medical College Hospital, Sylhet, Bangladesh:** Probhat Ranjan Dey MBBS, FCPS, MD, Nasreen Akhter MBBS, DGO, FCPS (from 2019)
  - **Jalalabad Ragib-Rabeya Medical College Hospital, Sylhet, Bangladesh:** Md. Abdus Sabur MBBS, FCPS (2017-2019), Mohammad Tarek Azad MBBS, DCH, MCPS, MD
  - ***Enam Medical College and Hospital, Savar, Dhaka, Bangladesh*:** Gulshan Ara MBBS, FCPS (2017-2019)**,** Shaheen Akter MBBS, MD, FCPS (2017-2019)
  - **Institute of Child and Maternal Health, Matuail, Dhaka, Bangladesh:** Md Mozibur Rahman MBBS, DCH, FCPS, Farida Yasmin MBBS, DGO, MCPS, FCPS
  - **Sylhet Women's Medical College Hospital, Sylhet, Bangladesh:** M. A. Matin MBBS, DCH, Shahana Ferdous Choudhury MBBS, FCPS

- **India site:**
    - **KLE Academy of Higher Education and Research, Jawaharlal Nehru Medical College, Belgaum, Karnataka, India:** Shivaprasad S Goudar MD, MHPE (Principal Investigator), Sangappa M Dhaded MD (Principal Investigator), Mrityunjay C Metgud MD, Yeshita V Pujar MD, Manjunath S Somannavar MD, Sunil S Vernekar MD, Veena R Herekar MD
    - **S Nijalingappa Medical College, Bagalkot, Karnataka, India:** Ashalata A Mallapur MD (Principal Investigator), Geetanjali M Katageri MS, Sumangala B Chikkamath MD, Bhuvaneshwari C Yelamali MD, Ramesh R Pol MD
    - **BLDE University's Shri B M Patil Medical College, Bijapur, Karnataka, India:** Shailaja R Bidri MD (Principal Investigator), Sangamesh S Mathapati MD, Preeti G Patil DNB, Mallanagouda M Patil MD, Muttappa R Gudadinni MD, Hidaytullah R Bijapure MD
    - **Srirama Chandra Bhanja Medical College, Cuttack, Odisha, India:** Sujata S Misra MD (from 2018) (Principal Investigator), Leena Das MD (from 2018), Saumya Nanda MD (from 2018), Rashmita B Nayak MD (from 2018), Bipsa Singh MD (from 2018)
- **Kenya site:**
    - **University of Nairobi, Nairobi, Kenya:** Zahida Qureshi MBBS, MMed (Principal Investigator), Fredrick Were MBChB, MMed, FNIC, PhD (Principal Investigator), Alfred Osoti MBChB, MMed, MPH, PhD, George Gwako MBChB, MMed, Ahmed Laving MBChB, MMed
    - **Kenyatta National Hospital, Nairobi, Kenya:** John Kinuthia MBChB, MMed, MPH (Principal Investigator)
    - **Coast Provincial General Hospital, Mombasa, Kenya:** Hafsa Mohamed MBChB, MMed (2018-2019), Nawal Aliyan MBChB, MMed (from 2019)
    - **Nakuru Level 5 Hospital, Nakuru, Kenya:** Adelaide Barassa MBChB, MMed, Elizabeth Kibaru MBChB, MMed (2018-2019)
    - **Kiambu level 5 Hospital, Kiambu, Kenya:** Margaret Mbuga MBChB, MMed, Lydia Thuranira MBChB, MMed (from 2019)
    - **Thika level 5 Hospital, Thika, Kenya:** Njoroge John Githua MBChB, MMed, Bernadine Lusweti MBChB, MMed, ESPE Fellow
- **Nigeria (Ibadan) site:**
    - **College of Medicine, University of Ibadan and University College Hospital, Ibadan, Nigeria:** Adejumoke Idowu Ayede MBBS, MSc., FMCPead, FRCPE (Principal Investigator), Olubukola Adeponle Adesina MBBS, FWACS, MSc (from 2019) (Principal Investigator),

Adegoke Gbadegesin Falade MBBS, MD, FMCPead, FRCPE, Atinuke Monsurat Agunloye MBBS, FMCR, FWACS, Oluwatosin Olaniyi Iyiola MBChB, MWACS

- **Kubwa General Hospital, Abuja, Nigeria:** Wilfred Sanni FWACS, Ifeyinwa Kate Ejinkeonye FWACP
- **Nyanya General Hospital, Abuja, Nigeria:** Hadiza Abdulaziz Idris MBBS, FMCOG, MRH, Chinyere Viola Okoli MBBS, FMCPead, MPH
- **State Specialist Hospital, Akure, Nigeria:** Theresa Azonima Irinyenikan MBBS, MPH, FMCOG, FWACS, Omolayo Adebukola Olubosede MBBS, MPH, FMCPaed, Olaseinde Bello MBChB, FWACP
- **Lagos Island Maternity Hospital, Lagos, Nigeria:** Olufemi Motunolani Omololu MBBS, FWACS, Olanike Abosede Olutekunbi MBBS, FWACP (from 2018)
- **Mother and Child Hospital, Akure, Nigeria:** Adesina Lawrence Akintan MBBS, FWACS, Olorunfemi Oludele Owa MBBS, MPA, FWACS (from 2018), Rosena Olubanke Oluwafemi MBChB, MPH, FWACP, Ireti Patricia Eniowo MBBS, FMCPaed (from 2018)
- **Lagos State University Teaching Hospital, Ikeja, Lagos, Nigeria:** Adetokunbo Olusegun Fabamwo MBBS, MSc, FMCOG, FWACS, Elizabeth Aruma Disu MBBCh, MSc., FWACPaed, Joy Onyinyechi Agbara MBBS, MSc, FMCOG, FWACS

- **Nigeria (Ile-Ife) site:**
  - **Obafemi Awolowo University, Ile-Ife, Nigeria:** Oluwafemi Kuti FWACS, FMCOG, FRCOG (Principal Investigator), Ebunoluwa Aderonke Adejuyigbe BSc, MBChB, FMCPaed (Principal Investigator), Henry Chineme Anyabolu MBBS, FWACP, Ibraheem Olayemi Awowole FWACS, FMCOG, MRCOG, Akintunde Olusegun Fehintola MBBS, FMCOG, MPH, Bankole Peter Kuti MBChB, FWACP, FMCPaed
  - **University of Benin, Benin City, Nigeria:** Adedapo Babatunde Anibaba Ande BSc, MBChB, FWACS, MPH, Ikechukwu Okonkwo MBBS, FWACP
  - **University of Ilorin, Ilorin, Nigeria:** Omotayo Adesiyun MBBS, FMCPaed, Hadijat Olaide Raji MBBS, MSc, FWACS (from 2019)
  - **University of Abuja, Gwagwalada, Federal Capital Territory, Nigeria:** Anthony Dennis Isah MBBS, FWACS, Eyinade Kudirat Olateju MBBS, FMCPaed
  - **Sacred Heart Hospital, Lantoro, Abeokuta, Nigeria:** Abiodun Olusanya MBCHB, FWACS, Olabisi Florence Dedeke MBBS, FWACPaed MPH
  - **Mother & Child Hospital, Ondo, Nigeria**: Lawal Oyeneyin FWACS, FMCOG, FICS, mni, Francis Bola Akinkunmi MBChB, FWACPaed

- **Pakistan site:**
    - **Aga Khan University, Karachi, Pakistan:** Shabina Ariff MBBS, FCPS, FCPS (Principal Investigator), Sajid Bashir Soofi MBBS, FCPS (Principal Investigator), Lumaan Sheikh MBBS, FCPS, MRCOG, FRCOG (Principal Investigator)
    - **Sheikh Zayed Medical College and Hospital Rahim Yar Khan, Pakistan:** Saima Zulfiqar MBBS, MCPS, FCPS, Sadia Omer MBBS, FCPS
    - **Liaquat University Hospital Hyderabad, Pakistan:** Raheel Sikandar MBBS, FCPS, Shazia Rani MBBS, FCPS, Salma Sheikh MBBS, DCH, MRCPCH, FRCPCH, PGPN
- **Data Management Team:**
    - **Centro Rosarino de Estudios Perinatales, Rosario, Argentina:** Daniel Giordano BSc, Hugo Gamerro BSc, Guillermo Carroli MD
- **Statistical Programming:**
    - **Statistika Consultoria, São Paulo, Brazil:** Jose Carvalho BSc, MSc., PhD
- **Technical Advisory Group:**
    - **University of Liverpool, Liverpool, United Kingdom**: James Neilson MD, FRCOG (Trial Steering Group chair)
    - **College of Medicine, University of Malawi, Blantyre, Malawi**: Elizabeth Molyneux FRCPCH, FRCEM, DSC h.c
    - **American University of Beirut, Lebanon**: Khalid Yunis MD, FAAP
    - **College of Health Sciences, University of Makerere, Kampala, Uganda:** Kidza Mugerwa MBChB, MMed
    - **Vardhman Mahavir Medical College and Associated Safdarjang Hospital, New Delhi**: Harish Kumar Chellani MD, DCH

Statistical methods

Sample size

We estimated the sample size on the basis of the primary outcome neonatal mortality at 28 completed days with a two-sided 5% significance level test and a power of 90%. A total of about 5,416 women are needed to detect a reduction of 15% or more from a 25% deaths to 21.3%, among neonates of women who were administered ACS at <34 weeks. With 10% loss to follow-up, we estimated that about 6,018 women had to be recruited.

For the composite possible maternal bacterial infection outcome, a non-inferiority hypothesis was used. A total sample size of 5,024 women are needed (including 10% loss to follow up) to demonstrate non-inferiority within that margin of 2.5% for the increase in the maternal infection outcome, assuming equal prevalence of 10% in the two arms, with a power of 80% and a significance level of 2.5%.

Statistical analysis

The primary analysis was performed according to the intention-to-treat principle based on all participants with outcome data available. The analysis of the three primary outcomes was adjusted for multiplicity. For the primary outcomes, fetal/neonatal mortality and maternal severe infection outcomes pertain to the enrolled population, whereas neonatal mortality pertains to liveborn neonates only.

We also conducted a secondary "per-protocol" analysis for the maternal primary outcome, as recommended for non-inferiority analyses, excluding women with protocol violations that might affect the primary outcome.

Baseline characteristics were compared between groups to detect imbalances in prognostic variables that could bias the results. Most study outcomes are binary variables. For this type of variables, the number of participants, number of missing values and percentages by group were reported. The intervention arm was compared against the control arm for the three primary outcomes using risk ratios with 95% confidence intervals. The statistical technique used to conduct tests and obtain confidence intervals was a logistic model with a binomial distribution and the log link to obtain relative risks. The stratifying variable study facility, a design variable, was included in the model, as well as a clustering feature for multiple births for neonatal outcomes. Separate models were fitted for each of the primary and secondary outcomes.

For continuous variables, the number of participants, the number of missing values, means and standard deviations or medians, quartiles and interquartile range (IQR) by group were reported. The intervention arm was compared against the control arm using mean or median differences and 95% confidence intervals. The statistical technique used to conduct tests and obtain confidence intervals for this type of variables was a general linear model including study facility in the model as stratifying variable, as well as a clustering feature for multiple births for neonatal outcomes.

All models were fitted using SAS Software version 9.4 (SAS Institute Inc., Cary, NC, USA).

We conducted the following planned stratified analyses:

- Planned preterm birth: yes vs. no
- Gestational age at first dose: 26 to <28 weeks vs. 28 to <32 weeks vs. 32 to <34 weeks
- Number of fetuses: single vs. multiple
- Study site: Bangladesh vs. India vs. Kenya vs. Nigeria (Ibadan) vs. Nigeria (Ile-Ife) vs. Pakistan
- Time from first dose to birth: 0 to 6h, >6 to 12h, >12 to 24h, >24h to 1 week, Over 1 week
- Mode of birth: vaginal birth vs.cesarean section
- Any use of tocolytics: yes vs. no

Data safety monitoring

A Data Safety Monitoring Board (DSMB) was appointed to monitor accruing trial data, in strict confidence, and three interim analysis were planned. The DSMB terms of reference were that they should inform the steering group chair if, in their view, there was proof beyond doubt that treatment with dexamethasone is indicated or contraindicated based on statistical considerations, practical issues, clinical considerations or external new information. The DSMB considered the Haybittle-Peto stopping rule on the primary infant mortality outcomes, as statistical guidance for their recommendation. Using this rule, a two-sided test of hypothesis to assess superiority of one of the groups (intervention or placebo) was conducted. If the result was significant at α=0.001, the DSMB would consider recommending stopping the trial for superiority of one of the groups.

Two interim analyses were conducted by both the trial statistician (blinded) and the DSMB statistician (unblinded on request) and results were presented at DSMB meetings. The DSMB could be unblinded to the study groups if and when needed. The first interim analysis was conducted

when 874 women and 972 infants (including 894 liveborn neonates) had been recruited and their complete data entered in the database. At their meeting on 19-20 November 2019, after review of 2304 women and 2536 infants (including 2337 liveborn neonates) with complete follow-up of primary outcomes, the DSMB decided to unblind the trial and recommended the trial to be stopped for mortality benefits, supported by evidence of a graded dose-response effect. Recruitment was stopped across all sites on 21 November 2019 and all ethics committees and regulatory authorities were informed of the decision to stop.

DSMB rationale for stopping the trial

The DSMB decided to recommend that the trial be stopped because they decided after lengthy debate that the evidence of benefit was so strong that they judged it unethical to continue.

They recognized that this was a deviation from the stopping rule. However, it was in line with Section 3.4.4 of the trial protocol, which specified that the DSMB decision to stop the trial following an interim analysis was to be guided not only by statistical considerations, but also by practical issues (adverse events, ease of treatments administration, unanticipated costs), as well as clinical considerations or external new information. Likewise, in Section 8.2 of the DSMB charter for the trial (**The role of formal statistical methods, specifically which methods will be used and whether they will be used as guidelines or rules**), it is stated that: "The statistical stopping rules should not be taken as the only criterion for a recommendation to stop the trial. Safety results from the trial as well as external information should be considered. A recommendation to discontinue recruitment, in all patients or in selected subgroups, will be made only if the result is likely to convince a broad range of clinicians, including those supporting the trial/s and the general clinical community."

The decision to stop the trial was driven by:

1) External new information from sheep studies became available during the conduct of the trial about strong effect of duration of fetal exposure to glucocorticoids on fetal lung maturation[1,2]
These studies concluded that the duration of materno-fetal glucocorticoid exposure, not total dose or peak drug exposure, is a key determinant for a sustained fetal lung maturation and antenatal glucocorticoid efficacy. Evidence of fetal lung maturation was observed with at least 24 hours of glucocorticoid exposure, with exposure of 48 hours providing more sustained effect.

On account of this external information, the DSMB decided to carry out a planned pre-specified sensitivity analysis excluding women giving birth less than 24 hours during their second interim

analysis and to include the findings in their decision making. This decision was made blinded to treatment allocation.

On completion of the second interim analysis of 2304 women and 2536 infants using the database closed in November 2019, the DSMB noted a clear evidence of reduction in both neonatal mortality and in any baby death, the two primary outcomes, in the dexamethasone intervention arm compared to the control (placebo) arm. At that time, the overall result was a relative reduction of 18% (95% CI: 5% to 29%; p=0.008) in neonatal death with dexamethasone, and a relative reduction of 13% (95% CI: 2% to 23%; p=0.02) in any baby death in the dexamethasone arm, compared to placebo. However, the results of the planned pre-specified sensitivity analysis on account of external new information described above, excluding women who delivered within 24 hours of the first injection of trial medication (whose babies would not be expected to benefit because of a short exposure to glucocorticoid) showed a 30% reduction in neonatal death (P=0.0017) and a 24% reduction in any baby death (P=0.0015). This analysis further showed that dexamethasone effects strengthened for both neonatal primary outcomes as women with varying degrees of shorter intervals between first injection and birth were excluded, reaching the z=3 level after those who could only have received one dose (i.e. up to 12 hours) are removed. These findings were indicative of graded dose-response relationship and efficacy of dexamethasone.

2) Considering the evidence from the trial in the context of the existing evidence of the benefits of antenatal glucocortioids (from the Cochrane review meta-analysis), well beyond the stopping boundary

While acknowledging the fact that the P-values from these analyses were very close to but did not strictly attain the 0.001 specified by the Haybittle Peto rule, the DSMB noted that these findings were consistent with the results of the Cochrane review (involving 7774 women and 8158 infants) that largely included studies from high-income countries, which showed overall reduction of 31% in neonatal death, and concluded that it would be unethical to further expose more women (and babies) to placebo given the existing body of knowledge from high-income setting. The DSMB was not only sensitive to these individual ethics but also considered the findings of these analyses convincing to influence policy and clinical practice (collective ethics), according to the DSMB charter.

Based on these considerations, the DSMB recommended that all recruitment be stopped, and this recommendation was unanimously accepted by the Technical Advisory Group, ACTION Trial Investigators, and WHO. The funder had no role in the deliberations and in the decision to stop the trial.

Assessing confounders and effect modifiers of neonatal primary outcomes

The effect of treatment, gestational age, time from first dose to birth and number of doses on the probability of baby death and neonatal death was assessed using a logistic model.

Variables:

- Response: any baby death or neonatal death
- Treatment (randomized): dexamethasone and placebo
- Site: (randomization was done within sites)
- Covariates: gestational age (weeks), time from first dose to birth (hours), number of doses.

Model

$$y = \log\left(\frac{p}{1-p}\right) = \mu + treat + site + exposure \cdot (treat) + exposure^2(treat) + ndoses(treat) + ga(treat)$$

where

A(B) means A within B,

p=proportion of events for binary neonatal outcome,

$$p = \frac{1}{1+e^{-y}}$$

y=logit for binary neonatal outcome (any baby death or neonatal death)

treat=treatment

exposure= time from first dose to birth (hours)

ndoses=number of doses

ga=gestational age at first injection (weeks)

site=study site

Gestational age at first injection was used instead of gestational age at birth because the latter is confounded with time from first dose to birth. The time interval between trial entry and birth is thus split in two non-overlapping time intervals (gestational age at first injection and time from first dose to birth).

Models were considered including terms for interactions, and the final model was selected excluding interaction terms that were not significant at 5%. Significance is assessed by p-values, in raw format and also expressed as logWorth, a logarithmic transformation of the P-value:

$$logWorth = -\log_{10}(p) = \log_{10}(1/p)$$

Goodness of fit of the model was assessed by the difference between the log-likelihood of the saturated model and that of the fitted model.

The effect of treatment was calculated in terms of relative risk (RR) from the model and plotted against time from first dose to birth by categories of gestational age at first injection.

Results

Neonatal death

The following table shows, for **the neonatal death outcome**, the significance for the different terms in the model described above. The most important effect by far is gestational age at first injection. Time from first dose to birth, study site, number of doses and treatment are significant at 1% level. The effects of gestational age, time from first dose to birth and number of doses are significantly different for each treatment.

Source	LogWorth	p-value
ga(treat)	97.784	0.00000
exposure(treat)	7.795	0.00000
site	4.187	0.00006
ndoses(treat)	3.042	0.00091
treat	1.873	0.01339
exposure*exposure(treat)	0.816	0.15267

The following table shows statistics of goodness of fit. The P-value for goodness of fit is 1, suggesting that the model fits the data well.

**Source**	**DF**	**-LogLikelihood**	**p-value**
Saturated model	2803	5.5452	
Fitted model	14	1141.4016	
Lack of fit	2789	1135.8564	1.0000

Any baby death

The following table shows, for **the baby death outcome**, the significance for the different terms in the model described above. The effects are very similar to those described for the neonatal death outcome.

Source	LogWorth	p-value
ga(treat)	116.999	0.00000
exposure(treat)	8.226	0.00000
site	3.438	0.00036
ndoses(treat)	2.632	0.00233
treat	1.073	0.08459
exposure*exposure(treat)	0.326	0.47224

The following table shows statistics of goodness of fit. The P-value for goodness of fit is 0.9785, suggesting that the model fits the data well.

**Source**	**DF**	**-LogLikelihood**	**p-value**
Saturated model	3028	6.9315	
Fitted model	14	1436.3927	
Lack of fit	3014	1429.4613	0.9785

Primary and secondary outcome definitions

PRIMARY OUTCOMES	OPERATIONAL DEFINITION AND MEASUREMENT
1. Neonatal death	Death of a live birth within 28 completed days of life.
2. Any baby death (stillbirth or neonatal death)	Any death of a fetus (post randomization) or death of a live birth within 28 completed days of life.
3. Possible maternal bacterial infection	Occurrence of maternal fever or clinically suspected or confirmed infection, for which therapeutic antibiotics were used.<br><br>*Suspected or confirmed infection could be an obstetric infection (chorioamnionitis, postpartum endometritis, or wound infection) or non-obstetric infection, as defined below. Captured during hospital admission/s only*
**SECONDARY OUTCOMES**	
A. For the neonate	
**A1. Mortality outcomes**	
1. Stillbirth	Any death of a fetus (post randomization).
2. Early neonatal death	Death of a live birth within 7 completed days of life.
**A2. Morbidity outcomes**	
3. Severe respiratory distress*‡	Clinical features are the presence of fast breathing (respiratory rate ≥ 70 breaths per minute) AND at least one of the following clinical signs:<br>1. Marked nasal flaring during inspiration,<br>2. Expiratory grunting audible with naked ear<br>3. Severe chest in drawing.<br>AND<br>SpO2 less than 90%, or use of supplemental oxygen.
4. Neonatal sepsis*	Defined as the presence of at least two (or more) of the following signs:<br>• Stopped feeding well<br>• Severe chest in-drawing<br>• Fever (body temperature of 38 °C or greater)<br>• Hypothermia (body temperature less than 35.5 °C)<br>• Movement only when stimulated or no movement at all<br>• Convulsions
5. Severe Intraventricular haemorrhage (sIVH)	Defined as a Papile's intraventricular hemorrhage classification grade 3 or 4, as per transcranial ultrasound assessment.<br><br>Liveborn neonates <34 weeks at birth will be routinely screened with transcranial ultrasound. Liveborn neonates ≥ 34 weeks at birth will receive transcranial ultrasound if indicated.<br><br>Transcranial ultrasound assessment will be performed at day 7 postnatal or discharge (if discharge occurs before 7 days after birth).
6. Neonatal hypoglycaemia*§	Neonatal hypoglycemia is defined as blood glucose measure less than 45 mg/dl (2.6mmol/l).

	All liveborn newborns in hospital will have glucose levels recorded at 6 and 36 hours (before feeding or IV fluids). Any documented hypoglycaemia will also be recorded.
7. Apgar score at 5 minutes	Assessment of neonatal vitality at 5 minutes after birth. Reported as Apgar score, and proportion of babies with Apgar <7.
B. For the Woman	
**B1. Mortality outcomes**	
8. Maternal death	Any maternal death in a trial participant, from time of randomization to 28 completed days postpartum.
**B2. Morbidity outcomes**	
9. Maternal fever	Maternal fever ≥38.0 C since randomization (on any one occasion, during hospital admission/s only).
10. Chorioamnionitis	Chorioamnionitis (suspected or confirmed) based on clinical assessment by obstetric care physician.<br><br>Clinical or laboratory features may include:<br>• Maternal fever ≥38.0 C<br>• Maternal and/or fetal tachycardia<br>• Purulent or foul smelling vaginal discharge<br>• Uterine tenderness<br>• Maternal leukocytosis<br>• Bacterial culture indicative of infection<br><br>measured during hospital admission only (from randomization until birth)
11. Postpartum endometritis	Postpartum endometritis (suspected or confirmed) based on clinical assessment by obstetric care physician.<br><br>Clinical or laboratory features may include:<br>• Maternal fever ≥38.0 C<br>• Maternal and/or fetal tachycardia<br>• Purulent or foul smelling vaginal discharge<br>• Uterine tenderness<br>• Maternal leukocytosis<br>• Bacterial culture indicative of infection<br><br>measured during hospital admission/s only
12. Wound infection	Infection of a wound or incision site (including perineal tear, episiotomy incision or cesarean section abdominal incision), suspected or confirmed by obstetric care physician<br><br>Measured during hospital admission/s only
13. Non-obstetric infection	Acute non-obstetric infection (suspected or confirmed) based on clinical assessment by obstetric care physician.<br><br>This includes:<br>• respiratory tract infection (including pneumonia, pharyngitis, sinusitis or similar)<br>• Urinary tract infection (excluding pyelonephritis)<br>• Pyelonephritis<br>• Acute cholecystitis<br>• Other systemic infection

	Malaria is specifically excluded from this outcome<br>Measured during hospital admission/s only
C. Process of care outcomes	
**C1. Measures of care given to neonate**	
14. Major neonatal resuscitation at birth	The use of positive pressure ventilation for more than one minute
15. Timing of breast milk feeding initiation*	Timing of initiation of breast milk feeding in hours after birth (breastfeeding, cup or tube feeding).
16. Time to full enteral feeding*	Timing to full enteral feeding (in days)
17. Use of oxygen therapy*	Defined as any use of oxygen therapy, using any method
18. Length of oxygen therapy*	This is defined as the total number of days of oxygen use during hospital stay. The total number of days will be counted, even if use was intermittent.
19. Use of continuous positive airway pressure (CPAP) ventilation*	Defined as any use of CPAP during admission to neonatal special care unit/ward
20. Length of use of continuous positive airway pressure (CPAP) ventilation*	Total number of days used will be counted, even if use is interrupted for hours or days.
21. Use of mechanical ventilation (MV)*	Any use of MV during admission
22. Length of use of mechanical ventilation (MV)*	Total number of days used will be counted, even if use is interrupted or intermittent
23. Any use of parenteral therapeutic antibiotic therapy for 5 or more days *	Any use of therapeutic antibiotics (intravenous or intramuscular) for 5 or more days, even if interrupted, excluding neonates who died before 5 completed days
24. Length of use of parenteral therapeutic antibiotic therapy*	Total number of days of use of parenteral antibiotic therapy
25. Use of surfactant treatment*	Any use of surfactant
26. Number of doses of surfactant treatment*	Total number of doses of surfactant treatment
**C2. Health service utilization (newborn)**	
27. Length of hospital stay after birth	Length of stay in hospital after birth in complete days (initial postnatal hospitalization only)
28. Admission to a special care unit (SCU)	Admission to special neonatal care unit or neonatal intensive care unit after birth (initial postnatal hospitalization only)

29. Length of admission to special care unit (days)	Length of admission to special neonatal care unit or neonatal intensive care unit in days
30. Newborn readmission for health care at facility	Any readmission to a health care at facility, for any reason.
31. Length of stay for newborn readmission	Length of readmission stay in facility in days
32. Number of newborn readmission for health care at facility	Number of readmissions for health care at facility, for any reason.
33. Cause of neonatal readmission for health care at facility	All causes of neonatal readmission to health care at facilities will be recorded as per clinical diagnosis
**C3. Measures of care given to woman**	
34. Therapeutic antibiotics	Therapeutic antibiotics for suspected or confirmed infection (obstetric or non-obstetric).<br>*Use of antibiotics for prophylaxis is not included in this outcome.*<br>Measured during hospital admission/s only
35. Number of days of therapeutic antibiotic use	Number of days of use of therapeutic antibiotics for suspected or confirmed infection (obstetric or non-obstetric).<br>Use of antibiotics for prophylaxis is not included in this outcome.<br>Measured during hospital admission/s only
36. Any antibiotic use	Any use of antibiotics in a randomized participant (maternal) while in facility (prophylactic or therapeutic)<br>Measured during hospital admission/s only
**C3. Health service utilization (woman)**	
37. Length of total maternal hospitalization for birth (days)	number of days which women are hospitalized for birth (i.e. the admission in which birth occurs).<br>Measured from day of admission to day of official discharge from facility, in days
38. Any postpartum maternal readmission to facility	Any postpartum readmission of the woman to hospital for any reason up to 28 completed days postpartum
39. Length of stay for postpartum maternal readmission	Length of readmission stay in facility in days
40. Number of maternal readmissions to facility	Number of postpartum readmissions of the woman to hospital for any reason up to 28 completed days postpartum

41. Cause of maternal readmission to facility	All causes of maternal readmission to hospital will be recorded as per clinical diagnosis
42. Any referral of woman to another facility for treatment of complications	Any referral of woman to another hospital for treatment of complications
Measures of compliance	
43. Compliance with study allocation	Defined as the proportion of women who complete the entire course, as per the allocation
44. Use of repeat course	Total number and proportion of women who received a repeat course of dexamethasone or placebo
45. Total number of treatment doses received	Total number of treatment (dexamethasone or placebo) doses received (initial and repeat)
46. Time from initiation of first dose until birth	Defined as the time from initiation of first dose (dexamethasone or placebo) to birth, measured in hours

** Measured during initial postnatal hospitalization only, until death, discharge or completed day 7 (whichever comes first); [‡]overall, and at 24 hours; [§] overall, and at 6 and 36 hours*

Supplementary figures and tables

Figure S1: Possible maternal bacterial infection by subgroups

Possible maternal bacterial infection

Subgroup	Dexamethasone	Placebo	Risk Ratio (95% CI)
	no. of events/total no.		
**Planned preterm birth**			
Yes	25/551	37/559	0.69 (0.42-1.12)
No	43/865	52/853	0.81 (0.55-1.20)
**Gestational age at first dose**			
26 to <28 weeks	10/136	12/123	0.79 (0.36-1.75)
28 to <32 weeks	33/657	48/666	0.68 (0.45-1.05)
32 to <34 weeks	24/598	29/592	0.82 (0.49-1.40)
**No. of fetus**			
Single	62/1255	85/1245	0.73 (0.53-0.99)
Multiple	5/137	4/138	1.23 (0.34-4.45)
**Study site**			
Bangladesh	4/198	6/195	0.66 (0.19-2.29)
India	9/287	24/292	0.38 (0.18-0.81)
Kenya	8/318	8/322	1.01 (0.38-2.66)
Nigeria (Ibadan)	21/234	21/230	0.98 (0.55-1.75)
Nigeria (Ile-Ife)	15/197	11/192	1.33 (0.63-2.82)
Pakistan	11/182	19/181	0.58 (0.28-1.18)
**Time from first dose to birth**			
0 to 6h	5/259	4/302	1.46 (0.40-5.39)
>6 to 12h	3/98	6/103	0.49 (0.13-1.89)
>12 to 24h	2/121	8/150	0.33 (0.07-1.50)
>24h to 1 week	24/490	32/405	0.61 (0.37-1.02)
Over 1 week	33/423	39/423	0.85 (0.55-1.31)
**Mode of birth**			
Vaginal birth	29/683	37/662	0.75 (0.47-1.21)
Cesarean section	34/663	49/690	0.73 (0.48-1.11)
**Tocolytic use***			
Yes	14/339	22/361	0.68 (0.35-1.29)
No	49/1020	65/1005	0.74 (0.52-1.06)
**All Participants**	**68/1416**	**89/1412**	**0.76 (0.56-1.03)**

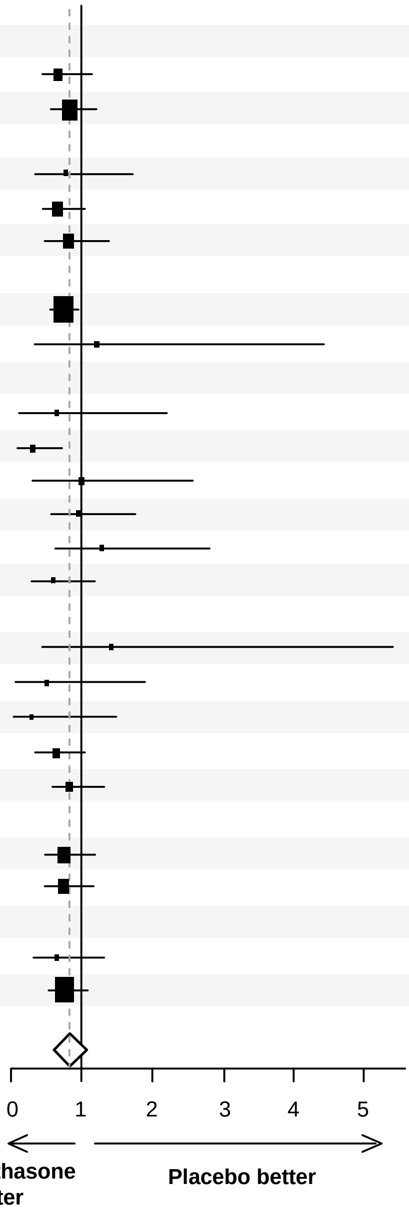

*Any tocolytic use before preterm birth

Figure S2. Relative risks of dexamethasone vs. placebo according to time from first dose to birth

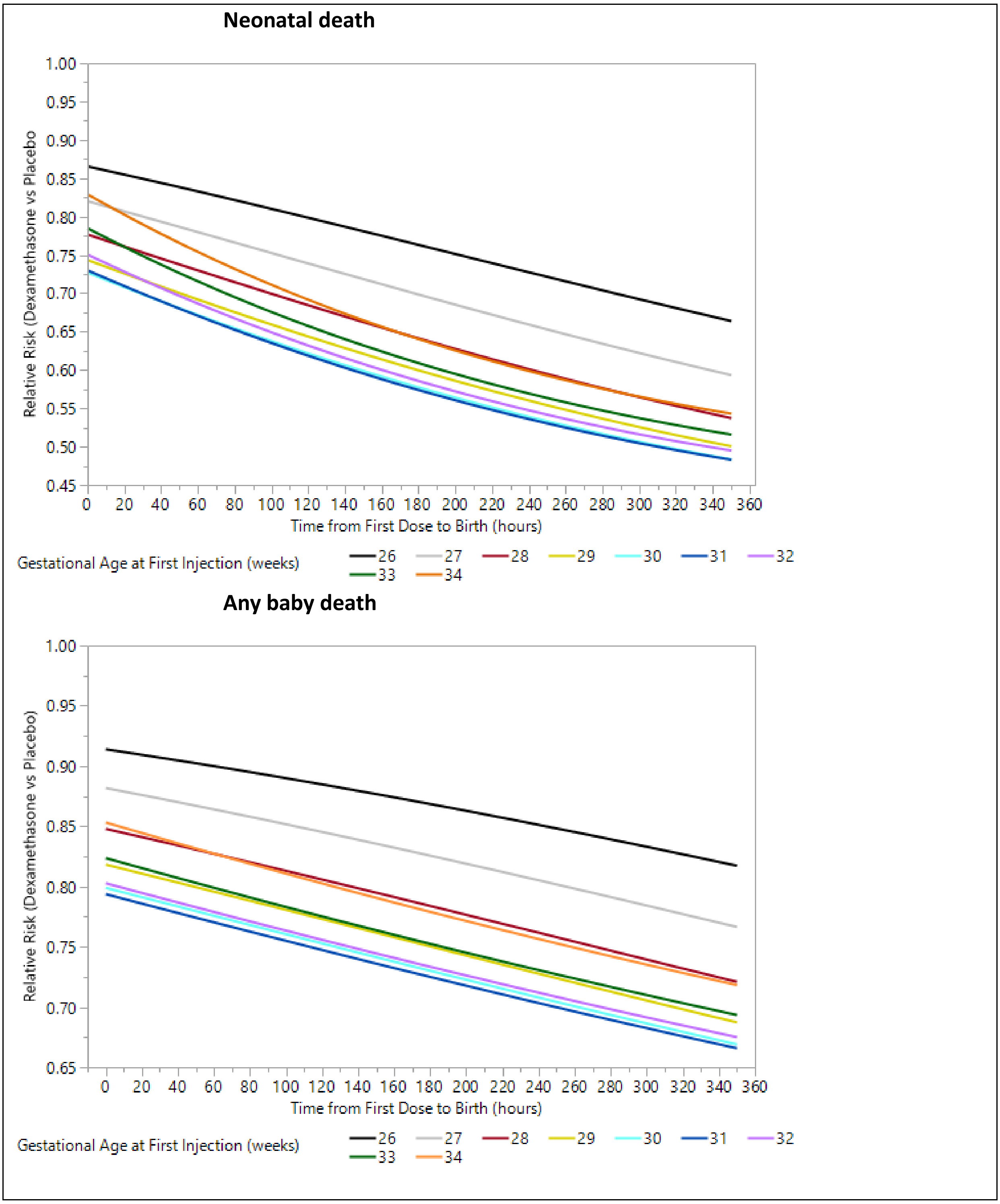

Figure S3 shows the relative risks (RR) as a function of the time from first dose to birth in hours, for different gestational ages at first injection, for the two neonatal primary outcomes. There is a trend for the relative risk to decrease with time from first dose to birth, suggesting that dexamethasone is

more protective as time of fetal exposure increases. It appears that the effect of dexamethasone is more protective as the gestational age at first injection increases from 26 until 32 weeks. However, this trend is not sustained as gestational age at first injection increase above 32 weeks. There might be confounding of time from first dose to birth with gestational age at birth that might mask or modify the effect of the intervention.

Table S1. Characteristics of ACTION-I trial hospitals

SITE	BANGLADESH					
**FACILITY**	**Facility 1**	**Facility 2**	**Facility 3**	**Facility 4**	**Facility 5**	**Facility 6**
Hospital location	Peri-urban	Peri-urban	Peri-urban	Peri-urban	Urban	Urban
Hospital level	Secondary	Tertiary	Tertiary	Tertiary	Tertiary	Tertiary
Number of births in 2016	1197	3000	5640	3624	3360	9180
Usual lower limit of gestational age for viability (i.e. active measures)	28 weeks 0 days	29 weeks 0 days	28 weeks 0 days	28 weeks 0 days	28 weeks 0 days	28 weeks 0 days
**OBSTETRIC CARE**						
All comprehensive obstetric care signal functions available	Yes	Yes	Yes	Yes	Yes	Yes
Number consultant obstetricians	9	7	14	24	7	12
Availability	Available during day time only	Available 24x7	Available during day time only	Available 24x7	Available 24x7	Available 24x7
What % of obstetricians are trained to perform ultrasound?	80%	100%	100%	80%	60%	5%
**Number of beds:**						
Admission area/s	55	46	100	180	47	42
Labour ward/s	5	2	4	30	27	42
Delivery ward/s	2	16	35	4	3	5
Postnatal ward/s	7	8	6	30	16	0
Maternal ICU	0	5	0	0	0	0

Maternal Special Care Unit	0	6	4	6	0	0
Post-operative ward/s	7	6	6	10	0	16
How soon after birth are women (without complications) routinely discharged?	24 hours	24 hours	8 hours	24 hours	24 hours	24 hours
**NEONATAL CARE**						
NICU available	No	Yes	Yes	Yes	Yes	No
If yes, how many beds:	-	20	50	6	12	-
Neonatal Special Care Unit available	No	No	Yes	Yes	No	No
If yes, how many beds:	-	-	85	4	0	-
**Thermal control in newborn ward:**						
N° of functioning incubators available:	1	10	5	3	3	4
N° of functioning radiant warmers available:	1	2	0	2	2	8
N° of functioning cradles available:	4	10	0	0	0	4
Shared use of the thermal control device	No	No	No	No	No	Yes
**Antibiotics administration**						
Intramuscular	No	No	No	Yes	Yes	Yes
Intravenous	Yes	Yes	Yes	Yes	Yes	Yes
Per oral	Yes	Yes	Yes	Yes	Yes	Yes

**Exogenous surfactant**	Not available	Always available when indicated	Not available	Always available when indicated	Not available	Not available
**Respiratory support**						
N° of functioning CPAP available:	1	3	4	3	3	0
N° of functioning Mechanical ventilators available:	0	2	0	6	2	0
**Number Consultant Neonatologists**	3	4	2	6	1	2
Availability	Available during day time only (after 8pm they are available over phone)	Available during day time only (after 8pm they are available over phone)	Available 24x7	Available 24x7	Available 24x7	Available during day time only (after 8pm they are available over phone)

**Number Consultant Paediatricians**	11		7	1	6	5	6
Availability	Available during day time only (after 8pm they are available over phone)		Available during day time only (after 8pm they are available over phone)	Available 24x7	Available 24x7	Available during day time only (after 8pm they are available over phone)	Available 24x7
**Diagnostic equipment**							
X-ray	Routinely available		Routinely available	Routinely available	Routinely available	Routinely available	Routinely available
Ultrasound for IVH	Not available		Routinely available	Routinely available	Routinely available	Routinely available	Available upon request

SITE	INDIA			
**FACILITY**	**Facility 1**	**Facility 2**	**Facility 3**	**Facility 4**
Hospital location	Urban	Urban	Urban	Urban
Hospital level	Tertiary	Tertiary	Tertiary	Tertiary
Number of births in 2016	6,116	4,012	10,082	2,405
Usual lower limit of gestational age for viability (i.e. active measures)	28 weeks 0 days	28 weeks 0 days	28 Weeks 0 Days	27 weeks 0 days
**OBSTETRIC CARE**				
All comprehensive obstetric care signal functions available	Yes	Yes	Yes	Yes
Number consultant obstetricians	18	18	28	8
Availability	Available 24x7	Available 24x7	Available 24x7	All available during day time. One available 24x7, all can be called if needed
What % of obstetricians are trained to perform ultrasound?	80%	90%	100%	80%
Number of beds:				
Admission area/s	36	60	40	7
Labour ward/s	12	8	12	8
Delivery ward/s	8	6	13	10
Postnatal ward/s	76	20	100	0
Maternal ICU	3	3	5	0
Maternal Special Care Unit	14	1	20	0
Post-operative ward/s	6	15	10	10
How soon after birth are women (without complications) routinely discharged?	72 hours	48 hours	24-48 hours	72 hours
**NEONATAL CARE**				
NICU available	Yes	Yes	Yes	Yes
If yes, how many beds:	14	30	22	40

Neonatal Special Care Unit available	No	Yes	Yes	No
If yes, how many beds:		30	24	
**Thermal control in newborn ward**:				
N° of functioning incubators available:	0	30	0	3
N° of functioning radiant warmers available:	14	30	10	40
N° of functioning cradles available:	0	0	0	10
Shared use of the thermal control device	No	No	No	No
**Antibiotics administration**				
Intramuscular	Yes	Yes	Yes	Yes
Intravenous	Yes	Yes	Yes	Yes
per oral	Yes	Yes	Yes	Yes
**Exogenous surfactant**	Available if parents can afford	Always available when indicated	Always available when indicated	Always available when indicated
**Respiratory support**				
N° of functioning CPAP available:	2	2	2	2
N° of functioning Mechanical ventilators available:	0	4	0	12
**Number Consultant Neonatologists**	2	0	0	1
Availability	0	N/A	N/A	1
**Number Consultant Paediatrician**	2	12	8	10
Availability	Available during day time only	Available 24x7	Available during day time only	Available 24x7
**Diagnostic equipment**				
X-ray	Available upon request	Routinely available	Available upon request	Routinely available
Ultrasound for IVH	Available upon request	Routinely available	Available upon request	Routinely available

SITE	KENYA			
**FACILITY**	**Facility 1**	**Facility 2**	**Facility 3**	**Facility 4**
Hospital location	Urban	Urban	Urban	Urban
Hospital level	Tertiary	Secondary	Secondary	Secondary
Number of births in 2016	10094	10544	7941	10334
Usual lower limit of gestational age for viability (i.e. active measures)	28 weeks 0 days	30 weeks	28 weeks 0 days	28 weeks 0 days
**OBSTETRIC CARE**				
All comprehensive obstetric care signal functions available	Yes	Yes	Yes	Yes
Number consultant obstetricians	2	2	3	2
Availability	Available 24x7	Available 24x7	Available 24x7	Available 24x7
What % of obstetricians are trained to perform ultrasound?	100%	50%	0	0
Number of beds:				
Admission area/s	5	2	7	1
Labour ward/s	14	35	3	12
Delivery ward/s	0	3	3	6
Postnatal ward/s	60	20	16	18
Maternal ICU	0	0	0	0
Maternal Special Care Unit	0	0	0	6
Post-operative ward/s	0	10	16	24
How soon after birth are women (without complications) routinely discharged?	24 hours	24 hours	24 hours	24 hours
**NEONATAL CARE**				
NICU available	Yes	No	Yes	Yes
If yes, how many beds:	1	-	16	40

Neonatal Special Care Unit available	Yes	No	No	Yes
If yes, how many beds:	44 cots 10 incubators			12
**Thermal control in newborn ward:**				
N° of functioning incubators available:	10	6	6	14
N° of functioning radiant warmers available:	2	2	4	0
N° of functioning cradles available:	0	10	5	10
Shared use of the thermal control device	Yes	Yes	Yes	Yes
**Antibiotics administration**				
Intramuscular	No	No	No	No
Intravenous	Yes	Yes	Yes	Yes
Per oral	Yes	Yes	Yes	Yes
**Exogenous surfactant**	Not available	Not available	Not available	Not available
**Respiratory support**				
N° of functioning CPAP available:	0	0	0	0
N° of functioning Mechanical ventilators available:	1	0	0	0
**Number Consultant Neonatologists**	0	0	0	0
Availability	-	-	-	-
**Number Consultant Paediatrician**	2	2	2	2
Availability	Available 24x7	Available 24x7	Available 24x7	Available 24x7
**Diagnostic equipment**				
X-ray	Routinely available	Routinely available	Routinely available	Available upon request
Ultrasound for IVH	Routinely available	Not available	Routinely available	Available upon request

SITE	NIGERIA-IBADAN						
**FACILITY**	**Facility 1**	**Facility 2**	**Facility 3**	**Facility 4**	**Facility 5**	**Facility 6**	**Facility 7**
Hospital location	Urban	Urban	Urban	Urban	Urban	Peri-urban	Peri-urban
Hospital level	Secondary	Tertiary	Secondary	Secondary	Tertiary	Secondary	Secondary
Number of births in 2016	3000	3000	2750	2000	2580	3000	2653
Usual lower limit of gestational age for viability (i.e. active measures)	26 weeks 0 days	28 weeks 0 days	26 weeks 0 days	26 weeks 0 days	26 weeks 0 days	26 weeks 0 days	26 weeks 0 days
**OBSTETRIC CARE**							
All comprehensive obstetric care signal functions available	yes	yes	yes	yes	yes	yes	yes
Number consultant obstetricians	8	10	2	5	20	2	3
Availability	Available 24x7	Available 24x7	Available 24x7	Available 24x7	Available 24x7	Available 24x7	Available 24x7
What % of obstetricians are trained to perform ultrasound?	100%	0%	100	60%	25%	100%	67%
Number of beds:							
Admission area/s	36	30	10	0	44	35	0
Labour ward/s	17	15	4	8	5	3	8
Delivery ward/s	8	26	4	14	5	3	0
Postnatal ward/s	62	30	25	26	46	15	6
Maternal ICU	7	0	0	4	0	4	0

Maternal Special Care Unit	8	6	1	10	8	3	0
Post-operative ward/s	14	26	15	26	10	19	14
How soon after birth are women (without complications) routinely discharged?	24 hours	24 hours	8 hours	24 hours	2 days	24 hours	6 hours
**NEONATAL CARE**							
NICU available	Yes	Yes	Yes	No	Yes	No	No
If yes, how many beds:	6	12	6	-	12	-	-
Neonatal Special Care Unit available	Yes	Yes	Yes	Yes	Yes	Yes	Yes
If yes, how many beds:	12	20	12	14	26	9	7
**Thermal control in newborn ward:**							
N° of functioning incubators available:	12	3	4	3	6	3	5
N° of functioning radiant warmers available:	3	3	3	4	3	2	3
N° of functioning cradles available:	10	1	15	14	16	4	4
Shared use of the thermal control device	No	No	No	Yes	Yes	Yes	No

**Antibiotics administration**							
Intramuscular	Yes	Yes	Yes	Yes	Yes	Yes	Yes
Intravenous	Yes	Yes	Yes	Yes	Yes	Yes	Yes
Per oral	Yes	Yes	Yes	Yes	Yes	Yes	Yes
**Exogenous surfactant**	Available for babies with severe illnesses	Not available	Not available	Not available	Not available	Not available	Not available
**Respiratory support**							
N° of functioning CPAP available:	3	3	2	2	3	1	2
N° of functioning Mechanical ventilators available:	0	0	0	0	0	0	0
**Number Consultant Neonatologists**	1	2	2	1	3	2	1
Availability	Available 24x7	Available 24x7	Available 24x7	Available 24x7	Available 24x7	Available 24x7	Available 24x7
**Number Consultant Paediatricians**	1	0	2	4	3	2	2
Availability	Available 24x7	-	Available 24x7	Available 24x7	Available 24x7	Available 24x7	Available 24x7
**Diagnostic equipment**							
X-ray	Routinely available	Routinely available	Routinely available	Routinely available	Not provided	Routinely available	Available upon request
Ultrasound for IVH	Routinely available	Routinely available	Routinely available	Routinely available	Not provided	Routinely available	Available upon request

SITE	NIGERIA- ILE IFE					
**FACILITY**	**Facility 1**	**Facility 2**	**Facility 3**	**Facility 4**	**Facility 5**	**Facility 6**
Hospital location	Urban	Peri-urban	Peri-urban	Urban	Urban	Urban
Hospital level	Tertiary	Tertiary	Secondary	Tertiary	Tertiary	Tertiary
Number of births in 2016	2256	1829	1590	2210	2056	1874
Usual lower limit of gestational age for viability (i.e. active measures)	27 weeks 0 days	26 weeks 0 days	27 weeks 0 days	26 weeks 0 days	26 weeks 0 days	24 weeks 0 days
**OBSTETRIC CARE**						
All comprehensive obstetric care signal functions available	yes	yes	yes	yes	yes	yes
Number consultant obstetricians	14	13	2	7	14	20
Availability	Available 24x7	Available 24x7	Available 24x7	Available 24x7	Available 24x7	Available 24x7
What % of obstetricians are trained to perform ultrasound?	100%	65%	50%	80%	100%	50%
Number of beds:						
Admission area/s	1	4	3	4	16	18
Labour ward/s	10	14	6	5	10	12
Delivery ward/s	10	8	6	0	4	8
Postnatal ward/s	30	49	42	16	32	Included in admission area
Maternal ICU	4	7	0	2	4	Included in General ICU 6 beds

Maternal Special Care Unit	0	8	2	1	0	Included in admission area and labour ward
Post-operative ward/s	30	34	21	11	25	Included in admission area
How soon after birth are women (without complications) routinely discharged?	24-48 hours	36-48 hours	48 hours	24 hours	24 - 48 hours	24 - 48 hours
**NEONATAL CARE**						
NICU available	No	No	No	Yes	Yes	Yes
If yes, how many beds:	-	-	-	8	25	N/A
Neonatal Special Care Unit available	Yes	Yes	Yes	Yes	Yes	Yes
If yes, how many beds:	32	33 cots, 15 incubators	22	15	25	50
**Thermal control in newborn ward:**						
N° of functioning incubators available:	13	15	6	4	6	21
N° of functioning radiant warmers available:	3	2	3	5	6	10
N° of functioning cradles available:	18	33	none	4	30	50
Shared use of the thermal control device	yes	yes	yes	no	sometimes	no
**Antibiotics administration**						
Intramuscular	yes	yes	yes	no	yes	yes
Intravenous	yes	yes	yes	yes	yes	yes
Per oral	yes	yes	yes	yes	yes	yes

**Exogenous surfactant**	Not available	Not available (unless patient procures it)	Available for babies with severe illnesses	Not available	Not available	Always available when indicated
**Respiratory support**						
N° of functioning CPAP available:	1	1	3	2	1	15
N° of functioning Mechanical ventilators available:	0	1	0	0	1	6
**Number Consultant Neonatologists**	2	2	2	2	3	4
Availability	9	Available 24x7	Available 24x7	Available 24x7	Available 24x7	Available 24x7
**Number Consultant Paediatricians**	9	14	2	1	15	20
Availability	Available 24x7	Available 24x7	Available 24x7	Available 24x7	Available 24x7	Available 24x7
**Diagnostic equipment**						
X-ray	Routinely available	Available upon request	Routinely available	Routinely available	Routinely available	Routinely available
Ultrasound for IVH	Routinely available	Available upon request	Routinely available	Available upon request	Routinely available	Available upon request

SITE	PAKISTAN	
**FACILITY**	**Facility 1**	**Facility 2**
Hospital location	Urban	Urban
Hospital level	Tertiary	Tertiary
Number of births in 2016	16245	15000
usual lower limit of gestational age for viability (i.e. active measures)	26 weeks	28 weeks 0 days
**OBSTETRIC CARE**		
All comprehensive obstetric care signal functions available	yes	yes
Number consultant obstetricians	40	18
Availability	Available 24x7	Available 24x7
What % of obstetricians are trained to perform ultrasound?	15%	70%
Number of beds:		
Admission area/s	72	8
Labour ward/s	6	32
Delivery ward/s	0	32
Postnatal ward/s	62	80
Maternal ICU	0	4
Maternal Special Care Unit	2	4
Post-operative ward/s	62	80
How soon after birth are women (without complications) routinely discharged?	6 to 12 hours	12 to 24 hours
**NEONATAL CARE**		
NICU available	No	Yes
If yes, how many beds:	-	16

Neonatal Special Care Unit available	Yes	Yes
If yes, how many beds:	20	10
**Thermal control in newborn ward:**		
N° of functioning incubators available:	7	8
N° of functioning radiant warmers available:	8	10
N° of functioning cradles available:	8	30
Shared use of the thermal control device	Yes	Yes
**Antibiotics administration**		
Intramuscular	No	No
Intravenous	Yes	Yes
Per oral	Yes	Yes
**Exogenous surfactant**	Not available	Not available
**Respiratory support**		
N° of functioning CPAP available:	0	4
N° of functioning Mechanical ventilators available:	0	8
**Number Consultant Neonatologists**	no	7
Availability	On call rosters	Available 24x7
**Number Consultant Paediatrician**	8	9
Availability	Available 24x7	Available 24x7
**Diagnostic equipment**		
X-ray	Routinely available	Routinely available
Ultrasound for IVH	Not available	Not available

Table S2. Characteristics of women at trial entry

Characteristic	Dexamethasone (N=1429)		Placebo (N=1423)	
**Clinical assessment of imminent preterm birth at trial entry – no. (%)**				
**Spontaneously-initiated preterm birth**	874	(61.2)	858	(60.3)
Preterm prelabour rupture of membranes	455	(31.8)	388	(27.3)
Spontaneous preterm labour	419	(29.3)	470	(33.0)
**Provider-initiated preterm birth**	555	(38.8)	565	(39.7)
**Gestational age at trial entry – no. (%)**				
26 weeks 0 days to 27 weeks 6 days	130	(9.1)	114	(8.0)
28 weeks 0 days to 31 weeks 6 days	654	(45.8)	679	(47.7)
32 weeks 0 days to 33 weeks 6 days	643	(45.0)	628	(44.1)
34 weeks 0 days to 36 weeks 0 days	2	(0.1)	2	(0.1)
**Mean (± SD) gestational age at trial entry**	30.8	(2.0)	30.7	(2.0)
**Maternal age (yr) – mean (SD)**	27.5	(5.8)	27.5	(5.9)
Missing – n (%)	1	(0.1)	0	(0.0)
**Educational level completed – no. (%)**				
No education	174	(12.2)	163	(11.5)
Primary education only	373	(26.1)	412	(29.0)
Secondary education only	549	(38.4)	501	(35.2)
Post-secondary/tertiary education	329	(23.0)	342	(24.0)
No answer	4	(0.3)	5	(0.4)
**Marital status – no. (%)**				
Married/Cohabiting	1380	(96.6)	1372	(96.4)
Single/Separated/Widowed/Divorced	49	(3.4)	51	(3.6)
**No. of fetuses in the current pregnancy – no. (%)**				
Single	1295	(90.6)	1290	(90.7)
Twin	125	(8.7)	129	(9.1)
Higher order multiples	9	(0.6)	4	(0.3)
**Parity**				
0	529	(37.0)	549	(38.6)
1-2	646	(45.2)	630	(44.3)
3-4	217	(15.2)	195	(13.7)
5 or more	37	(2.6)	49	(3.4)
**History of preterm birth – no. (%) ***				
Yes	177	(12.4)	188	(13.2)
Unknown	28	(2.0)	21	(1.5)
**Maternal weight (kg) – mean (SD)**	65.4	(15.9)	64.2	(15.2)
Missing – n (%)	71	(5.0)	70	(4.9)

**Maternal height (cm) – mean (SD)**	156.0	(7.7)	155.7	(7.6)
Missing – n (%)	102	(7.1)	90	(6.3)
**Maternal midarm circumference (cm) – mean (SD)**	28.3	(4.9)	28.1	(4.9)
Missing – n (%)	53	(3.7)	61	(4.3)
**Medical conditions currently present – no. (%) ****				
Chronic hypertension	64	(4.5)	71	(5.0)
Diabetes mellitus (non-gestational)	13	(0.9)	14	(1.0)
HIV or AIDS	33	(2.3)	32	(2.2)
Tuberculosis	1	(0.1)	2	(0.1)
Pyelonephritis	5	(0.3)	13	(0.9)
Anaemia (hematocrit ≤26% or haemoglobin ≤9g/dL)	100	(7.0)	128	(9.0)
Malaria	48	(3.4)	55	(3.9)
**Obstetric conditions currently present – no. (%) ****				
Gestational diabetes	22	(1.5)	15	(1.1)
Pre-eclampsia or eclampsia	275	(19.2)	326	(22.9)
Gestational hypertension (excl. preeclampsia or eclampsia)	75	(5.2)	68	(4.8)
Oligohydramnios (known or suspected)	336	(23.5)	310	(21.8)
Polyhydramnios (known or suspected)	19	(1.3)	30	(2.1)
Intrauterine growth restriction (known or suspected)	94	(6.6)	95	(6.7)
Abruptio placentae	49	(3.4)	40	(2.8)
Placenta praevia	115	(8.0)	110	(7.7)
Other obstetric hemorrhage	66	(4.6)	42	(3.0)
No obstetric condition	616	(43.1)	592	(41.6)
**First date of last menstrual period known – no. (%)**				
Certain	844	(59.1)	826	(58.0)
Uncertain	173	(12.1)	166	(11.7)
Unknown	412	(28.8)	431	(30.3)
**Trimester of pregnancy when ultrasound for gestational age estimate was performed – no. (%)**				
1st trimester (up to 13 weeks 6 days)	156	(10.9)	147	(10.3)
2nd trimester (14 weeks 0 days to 27 weeks 6 days)	344	(24.1)	329	(23.1)
3rd trimester (28 weeks 0 days and beyond)	929	(65.0)	947	(66.5)
**Medication administered prior to randomization – no. (%)**				
Tocolytic	251	(17.6)	267	(18.8)
Magnesium sulfate for neuroprotection	141	(9.9)	179	(12.6)

**Only among women with a previous pregnancy. **Women may have had more than one condition. There was no significant difference between treatment groups at an experimentwise error rate of 5%.*

Table S3. Primary outcomes with multiple imputation of missing values*

Primary outcome	RR	Lower 95% CI	Upper 95% CI	P-value[§]
Neonatal death	0.8377	0.7224	0.9714	0.0193
Any baby death	0.8821	0.7811	0.9960	0.0438
Possible maternal bacterial infection	0.7598	0.5605	1.0302	0.0007

**20 imputations; [§] P-value for superiority for neonatal death and any baby death, and P-value for non-inferiority for possible maternal bacterial infection*

Table S4. Cause-specific neonatal mortality

Final cause of death	Dexamethasone (N=1417)	Placebo (N=1406)	Relative risk (95% CI)
Perinatal asphyxia – no. (%)	61 (4.3)	78 (5.5)	0.78 (0.56-1.07)
Respiratory distress syndrome – no. (%)	113 (8.0)	156 (11.1)	0.72 (0.57-0.90)
Neonatal sepsis – no. (%)	77 (5.4)	74 (5.3)	1.03 (0.76-1.41)
Other specific causes – no. (%)	18 (1.3)	12 (0.9)	1.49 (0.73-3.16)
Indeterminate – no. (%)	9 (0.6)	11 (0.8)	0.81 (0.33-1.96)

Table S5. Other secondary maternal and neonatal outcomes

Neonatal outcomes	Dexamethasone		Placebo		Mean or Median Difference (95% CI) [§]
	N	Mean (± SD) or Median (IQR)	N	Mean (± SD) or Median (IQR)	
Mean birth weight* – g	1495	1819 (623)	1482	1805 (624)	14.47 (-30.36 to 59.29)
Mean head circumference* – cm	1388	30 (3)	1378	30 (3)	0.10 (-0.12 to 0.32)
Mean body length* – cm	1387	42 (5)	1379	42 (5)	0.07 (-0.29 to 0.42)
Median gestational age at birth* – weeks	1544	33 (31-34)	1526	33 (31-34)	0.00 (-0.19 to 0.20)
Median duration of oxygen therapy – hours	726	36 (18-96)	756	48 (12-93)	-12.00 (-15.59 to -8.42)
Median duration of CPAP ventilation – hours	265	48 (24-96)	337	48 (24-84)	0.00 (-8.38 to 8.38)
Median duration of use of mechanical ventilation – hours	83	18 (12-48)	103	18 (12-60)	0.00 (-6.84 to 6.84)
Median duration of parenteral therapeutic antibiotic use – hours	864	144 (63-168)	894	132 (48-168)	11.85 (2.17 to 21.53)
Median length of hospital stay after birth – days	1320	8 (3-17)	1301	8 (3-17)	0.17 (-0.58 to 0.92)
Median duration of admission to special care unit – hours	905	168 (72-168)	897	162 (60-168)	6.00 (-4.99 to 16.99)
Median time until breast milk feeding initiation – hours	1126	24 (2-60)	1049	24 (2-60)	-0.14 (-4.02 to 3.73)

Median time to full enteral feeding – hours	667	12 (6-72)	628	12 (6-84)	0.00 (-0.20 to 0.20)
Median number of newborn readmission	39	1 (1-1)	48	1 (1-1)	-
Median length of stay during newborn readmission – days	37	5 (3-7)	37	4 (3-6)	1.00 (-1.13 to 3.13)
**Maternal outcomes**					
Median number of days of therapeutic antibiotic use – days	64	4 (1-6.5)	81	5 (2-7)	-1.40 (-2.92 to 0.13)
Median length of total maternal hospitalization for birth – days	1323	8 (4-20)	1322	8 (4-19)	0.30 (-0.54 to 1.15)
Median length of maternal re-admission – days	13	5 (3-11)	13	4 (1-9)	0.00 (-8.60 to 8.60)

**All babies were assessed, outcome not prespecified; [§]Adjusted for study site; Median number of doses of surfactant not presented because few participants received surfactant*

Table S6. Other secondary maternal and neonatal outcomes (contd.)

Outcome	Dexamethasone<br>n/N (%)	Placebo<br>n/N (%)	Relative risk (95% CI)
**Neonatal outcome**			
Apgar score <7 at 5 minutes	276/1359 (20.3)	293/1368 (21.4)	0.95 (0.82-1.10)
Use of surfactant*	9/1284 (0.7)	18/1264 (1.4)	0.49 (0.22-1.08)
Newborn readmission for care	39/1429 (2.7)	48/1413 (3.4)	0.81 (0.53-1.25)
**Maternal outcome**			
Postpartum readmission	14/1429 (1.0)	13/1423 (0.9)	1.07 (0.50-2.26)

** Measured during initial postnatal hospitalization only, until death, discharge or completed day 7 (whichever came first)*

Table S7. Adverse events

Adverse events	Dexamethasone	Placebo	Total
**Maternal adverse events**			
Antepartum haemorrhage	0	2	2
Dyspnea	0	1	1
Gastrointestinal upset	1	0	1
Hyperglycemia	0	1	1
Leucocytosis	0	1	1
Migraine (unspecified)	1	1	2
Postpartum haemorrhage	3	3	6
Pyrexia (unspecified)	0	1	1
Seizure	2	0	2
**Total**	**7**	**10**	**17**
**Maternal serious adverse events**			
Antepartum haemorrhage	1	2	3
Cerebrovascular accident	1	0	1
Dyspnea	0	1	1
Intrapartum hemorrhage	1	0	1
Maternal death*	5	4	9
Pleural effusion	0	1	1
Postpartum haemorrhage	4	4	8
Seizure	2	2	4
Uterine rupture	2	0	2
Wound hematoma	0	2	2
**Total**	**16**	**16**	**32**
**Neonatal adverse events***			
Birth asphyxia	1	1	2
Neonatal death	4	1	5
Neonatal sepsis	0	1	1
**Total**	**5**	**3**	**8**

**Also captured as part of secondary outcome measures*

Summary of the procedures to determine the final cause of neonatal death

An exercise was undertaken to determine the final single cause of neonatal death in the trial. Neonatal death is reported in the perinatal cause of death (PCD)form for all deaths that occurred in the facility and in the verbal autopsy form for deaths that occur outside the study facilities. The WHO Newborn Health team reviewed all 609 neonatal deaths based on the forms completed at each site. Each neonatal death was assigned one underlying cause of death based on the following processes:

- All causes of death were classified into one of the following main causes of death: respiratory distress syndrome, neonatal sepsis, perinatal asphyxia, other specific cause or indeterminate.
- Verbal autopsies were reviewed where PCD form was not available and a cause of death was assigned.
- ICD principles were followed in assigning the cause of death.
- Where no valid cause of death was available in the PCD form, all available forms for the infant were reviewed to assign a valid cause of death.
- The site-specific list of cause of death was reviewed by the neonatal Principal Investigators at the respective sites and compared with the source documents. The changes suggested by the PIs were made.

The list of final cause of death was shared with the statistical analysis team to determine cause specific mortality by study groups.

Procedures relating to ultrasound assessments

All participating hospitals were provided with the following ultrasound equipment:

- 1 x Philips HD5 ultrasound system
- 3 x probes – transabdominal, transcranial and intravaginal
- 1 x Uninterruptible Power Supply (UPS) device

This equipment was expressly for the purposes of facilitating assessment and recruitment of women to the ACTION trials and assessment of intraventricular haemorrhage in neonates (hereafter referred to as the ACTION Trial ultrasound systems). It was intended to augment existing ultrasound systems at participating hospitals, and (to the extent possible) minimize ultrasound access issues for trial participants.

Obstetric ultrasound for gestational age assessment

There are several considerations for performance of dating ultrasounds in low resource settings:

- Accurate estimated gestational age (EGA)/expected delivery date (EDD) assignment is limited by multiple factors:
  - Late initiation of antenatal care;
  - Uncertain last menstrual period;
  - No prior ultrasound evaluation (estimated gestational age has been assigned by a referring care provider based on fundal height only);
  - Third trimester fetal biometric variance (+/- 21 days at >28 weeks estimated gestational age);
  - Prior scans performed by sonographers outside of the hospital with varying/unknown levels of experience or expertise; and
  - Use of biometric nomograms derived from a different (often higher resource) populations.

Furthermore, many tertiary-level maternity facilities in low-resource countries do not always have routine or 24/7 access to obstetric ultrasound services.

In order to optimize the assessment of gestational age in routine care settings, the following procedures were developed and applied:

- For women to be eligible for the trial, the gestational age must be based on the earliest available obstetric ultrasound of reasonable quality. In the event an ultrasound was available from earlier in the pregnancy, the obstetric physician determined whether this ultrasound was of acceptable quality. If it was not available (or no ultrasound assessment was available), a dating ultrasound was performed at the participating hospital.
- The study Manual of Operations provided a gestational age estimation algorithm that was adapted from American College of Obstetrics and Gynaecology (ACOG) Committee Opinion on Method for Estimating Due Date (October 2014).[3] These procedures were reviewed by two independent experts from the International Society of Ultrasound in Obstetrics and Gynaecology (ISUOG) (Dr Lynn Coppola and Dr Sandhya Maranna).
- Individuals at participating hospitals who were involved in performing obstetric ultrasound (varied by site, but generally involved ultrasonographers, radiologists and/or obstetricians)

underwent a standardized training provided by an ISUOG expert trainer (LC or SM). This training included use of ISUOG teaching modules as well as hands-on practice. Completion of 3 to 5 obstetric ultrasounds of acceptable quality was required to demonstrate proficiency.

The following measures were implemented for quality assurance:

- During the trial, the nominated Lead for obstetric ultrasound assessment at each hospital or study site conducted periodic internal peer-review of ultrasound scans performed, as well as any refresher training on an as-needed basis.
- For those women where the ACTION Trial ultrasound system was used to identify the gestational age, scans were digitally saved (using anonymized participant ID numbers) and logged in a standard logbook. The Manual of Operations pre-specified that approximately 5% of saved scans would be randomly sampled for quality assurance purposes.
- A random sample of scans for 175 participants (6.1% of the 2852 women randomized) were selected, reviewed and scored by an ISUOG expert. The scoresheet was pre-designed to assess whether the scan had been performed correctly from a technical standpoint, and was based on criteria of Salomon et al [4]. Based on available images and scores, scans were rated by the ISUOG expert as "acceptable" or "not acceptable" to be utilized by the sites for accurate estimation of gestational age.
- This sample was not evenly distributed across countries, as some hospitals (particularly those in India and Bangladesh) had a high proportion of women who had a dating ultrasound from the first trimester of pregnancy.
- Images of sufficient quality were available for 156 participants cases (5.5% of 2852 randomized women). Of these, 145 were rated "acceptable" (93%) and 11 were not acceptable.

Neonatal transcranial ultrasound intraventricular haemorrhage assessment

- **Equipment/Machine:** Philips HD5 scanner with a sector probe (5-8 MHz)
- **Protocol for obtaining neonatal CUS**: Transcranial ultrasound was performed routinely for newborns delivered at < 34 weeks by a trained provider at 7 days postnatal age or discharge, whichever occurred first. For babies born at ≥ 34 weeks, transcranial ultrasound was performed only when specifically requested by a clinician.
- **Presence and grading of intraventricular hemorrhage (IVH)**: The presence of IVH and its grading was evaluated as below:

- Any echogenicity at the level of caudothalamic groove (extending anterior to Foramen of Munro) is suggestive of IVH
- IVH was graded according to the grading proposed by *Papile* given below
    - Grade 1 – Sub-ependymal haemorrhage without ventricular extension
    - Grade 2 – Intraventricular Haemorrhage without ventricular dilatation
    - Grade 3 – Intraventricular Haemorrhage with ventricular dilatation
    - Grade 4 – Intraventricular haemorrhage with associated parenchymal involvement

- **Data/record maintenance:** The CUS scans at each site were digitally saved using anonymized participant ID numbers and logged in a standard logbook at each site.
- **Training and quality assurance:** The following measures were implemented for quality assurance:
    - Standard operating procedures were developed describing the CUS technique including the views required and other technical requirements, interpretation and grading of IVH.
    - Prior to trial initiation, all site sonologists were trained on standard operating procedures and interpretation and reporting by an expert. Around 70 staff were trained across all sites (including neonatologists, radiologists and sonologists, though staff cadre varied by site). The training involved a presentation on the basics of ultrasonography, hardware, CUS techniques and interpretation. The trainees were then trained on neonates under the supervision of the expert.
    - A sample of all positive scans (grade 1-4; as reported by sites) and a 5% random sample of all negative scans (grade 0; as reported by sites) were reviewed and graded independently by an external expert, blinded to the grading reported by sites. Any discrepancies in grading between the site sonologists and the external expert were reviewed and resolved by mutual discussion between the two.
    - Images were available for 108 of 137 (79%) positive scans and for 58 of 65 (89%) randomly selected negative scans:
        - Severe (grade 3-4), n=15 available of total 17 (88%): 5 graded same, 1 “downgraded” by expert but site maintained as “severe”
        - Non-severe (grade 1-2), n=93 available of total 120 (78%): 3 “upgraded” by expert (3%)
        - No IVH (grade 0), n= 65 available of total 65 (89%): all graded same by expert

References

1. Kemp MW, Saito M, Usuda H, et al. The efficacy of antenatal steroid therapy is dependent on the duration of low-concentration fetal exposure: evidence from a sheep model of pregnancy. Am J Obstet Gynecol 2018;219:301 e1- e16.

2. Schmidt AF, Kemp MW, Milad M, et al. Oral dosing for antenatal corticosteroids in the Rhesus macaque. PLoS One 2019;14:e0222817.

3. Committee on Obstetric Practice and the Society for Maternal-Fetal Medicine. Committee Opinion No 700: Methods for Estimating the Due Date. Obstet Gynecol 2017;129:e150-e4.

4. Salomon LJ, Bernard JP, Duyme M, Doris B, Mas N, Ville Y. Feasibility and reproducibility of an image-scoring method for quality control of fetal biometry in the second trimester. Ultrasound Obstet Gynecol 2006;27:34-40.
